# Inborn Errors of Purine Salvage and Catabolism

**DOI:** 10.3390/metabo13070787

**Published:** 2023-06-24

**Authors:** Marcella Camici, Mercedes Garcia-Gil, Simone Allegrini, Rossana Pesi, Giulia Bernardini, Vanna Micheli, Maria Grazia Tozzi

**Affiliations:** 1 Unità di Biochimica, Dipartimento di Biologia, Università di Pisa, Via San Zeno 51, 56127 Pisa, Italy; marcella.camici@unipi.it (M.C.); rossana.pesi@unipi.it (R.P.); maria.grazia.tozzi@unipi.it (M.G.T.); 2 Unità di Fisiologia Generale, Dipartimento di Biologia, Università di Pisa, Via San Zeno 31, 56127 Pisa, Italy; mercedes.garcia@unipi.it; 3 CISUP, Centro per l’Integrazione Della Strumentazione Dell’Università di Pisa, 56127 Pisa, Italy; 4 Centro di Ricerca Interdipartimentale Nutrafood “Nutraceuticals and Food for Health”, Università di Pisa, 56126 Pisa, Italy; 5 Dipartimento di Biotecnologie, Chimica e Farmacia, Università di Siena, Via A. Moro 2, 53100 Siena, Italy; giulia.bernardini@unisi.it (G.B.); vannamiche@gmail.com (V.M.); 6 LND Famiglie Italiane ODV-Via Giovanetti 15-20, 16149 Genova, Italy

**Keywords:** inborn errors, purine catabolism, purine salvage, neurological syndromes, metabolism, uric acid, neurological disorders, metabolites

## Abstract

Cellular purine nucleotides derive mainly from de novo synthesis or nucleic acid turnover and, only marginally, from dietary intake. They are subjected to catabolism, eventually forming uric acid in humans, while bases and nucleosides may be converted back to nucleotides through the salvage pathways. Inborn errors of the purine salvage pathway and catabolism have been described by several researchers and are usually referred to as rare diseases. Since purine compounds play a fundamental role, it is not surprising that their dysmetabolism is accompanied by devastating symptoms. Nevertheless, some of these manifestations are unexpected and, so far, have no explanation or therapy. Herein, we describe several known inborn errors of purine metabolism, highlighting their unexplained pathological aspects. Our intent is to offer new points of view on this topic and suggest diagnostic tools that may possibly indicate to clinicians that the inborn errors of purine metabolism may not be very rare diseases after all.

## 1. Introduction

Nucleotides and nucleosides are present in all kinds of cells as intermediates of the synthesis or catabolism of a plethora of compounds ranging from nucleic acids to chemical energy preserving and donor molecules, coenzymes, and signaling molecules. Therefore, the enzymes involved in their synthesis and catabolism are fundamental for cell life. Usually, the rates of purine synthesis and salvage are strictly regulated to produce enough purine compounds to sustain life [1]. All the exceeding compounds and most of the purines present in food are catabolized into uric acid (UA). In fact, it was long ago demonstrated that the intestinal epithelial cells efficiently catabolize the dietary nucleosides into the nucleobases, which are eventually converted into UA and released in blood [2,3], while ribose-1-phosphate can be utilized for energy production [4]. UA is maintained in blood at a concentration close to the limit of solubility [5], where it probably plays a positive function as an antioxidant but also as a regulatory compound in lipid and carbohydrate catabolism [6]. Since UA is the final product of purine catabolism in humans, it is not surprising that fluctuations in the rate of purine synthesis or catabolism often reflect on its hematic and urinary concentration [7,8,9].

**Figure 1:**
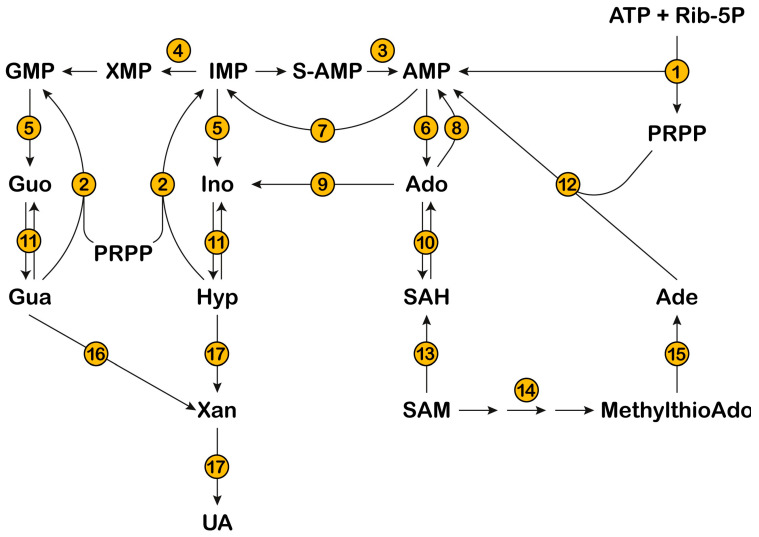
The metabolic scheme depicts the pathways involved in the catabolism and salvage of purine nucleotides. Inosine 5′-monophosphate (IMP) represents the branching point from which both guanylate and adenylate pools are generated. IMP dehydrogenase (4) converts IMP into xanthosine monophosphate (XMP), and adenylosuccinate lyase (3) converts succinyl-adenosine 5-monophosphate (S-AMP) into AMP. Purine nucleotides are converted to the corresponding nucleosides by 5′-nucleotidases (cytosolic 5′-nucleotidase I, 5, and cytosolic 5′-nucleotidase II, 6). Guanosine (Guo) and Inosine (Ino) are phosphorolytically cleaved into the bases guanine (Gua) and hypoxanthine (Hyp), through purine nucleoside phosphorylase (11). Adenosine (Ado) is deaminated to Ino by adenosine deaminase (9). Gua, Hyp, and Adenine (Ade) can be salvaged to the corresponding nucleotides by phosphoribosyltransferases (hypoxanthine-guanine phosphoribosyltransferase, 2, and adenine phosphoribosyltransferase, 12). Phosphoribosylpyrophospate (PRPP), necessary for the salvage of purine bases, is generated by phosphoribosylpyrophosphate synthetase (1), starting from ATP and ribose-5-phosphate (Rib-5-P). Ado can be generated from S-adenosylmethionine (SAM), through methyltransferases (13) and S-adenosylhomocysteine (SAH) hydrolase (10). Ado can be converted into AMP by adenosine kinase (8). Ade can be generated from SAM, through the polyamine synthesis pathway (14) and methylthioAdo phosphorylase (15). AMP is deaminated to IMP by AMP deaminase (7) and Gua is converted to xanthine (Xan) by guanase (16). Eventually, purine bases are converted to uric acid (UA) through xanthine oxidoreductase (17).

This review focuses on the inborn errors of enzymes depicted in Figure 1, which catalyze the steps involved in the salvage and catabolism pathways of purine nucleotides. Mutations of these enzymes lead, in most cases, to severe and rare disorders, mostly characterized by a complex combination of both metabolic and neurological symptoms [8,9,10]. The unexplained aspects stemming from enzyme mutations are mainly examined, while enzyme alterations that only yield well understood metabolic consequences are not described. The metabolic consequences are related to the accumulation of metabolites, which, in most cases, have a direct impact on kidney functions. For example, the accumulation of UA, due to a lack of hypoxanthine-guanine phosphoribosyltransferase (HPRT) (enzyme n.2, Figure 1) or the superactivity of phosphoribosylpyrophosphate synthetase (PRPS) (enzyme n.1, Figure 1) may lead to the formation of urate stones [11]. For the same reason, xanthine oxidoreductase (XOD) deficiency (enzyme n.17, Figure 1) leads to the accumulation of xanthine, which is responsible for the formation of stones and possibly of xanthine deposits in muscle [12]. Moreover, adenine phosphoribosyltransferase (APRT) deficiency (enzyme n.12, Figure 1) causes an accumulation of adenine, which, once converted by XOD to 2,8-dihydroxyadenine crystallizes in urine, forms stones in the kidneys and urinary tract [13]. DeoxyATP (dATP) and deoxyGTP (dGTP), which accumulate in cases of deficiencies of adenosine deaminase (ADA) (enzyme n.9, Figure 1) and purine nucleoside phosphorylase (PNP) (enzyme n.11, Figure 1), respectively, exert a cytotoxic effect, due to the dysregulation of ribonucleotide reductase, especially in cells of the immune system, which are particularly sensitive to deoxynucleotide (dNT) imbalance [14]. Neurological symptoms are more elusive and their mechanistic basis is difficult to understand. Inborn errors affecting enzymes, such as ADA, PNP, HPRT, and PRPS, besides the well-known metabolic consequences, also bring about severe neurological symptoms, which may arise later or, in some cases, before the metabolic ones. As underlined in the review, neurological impairment arises mainly from a defect in neurodevelopment; therefore, an early intervention would be advisable to avoid or attenuate the noxious effects of the disorder. Unfortunately, since inborn errors of purine metabolism are considered rare diseases with no effective therapy, newborn screening is not a normal practice. The extreme or presumed rarity of the above diseases has also reduced investigations to a very limited number of cases. Despite the enormous work undertaken to unravel the signaling pathways underlying the neurological manifestations, many aspects still remain obscure. Starting from this consideration, the aim of this review is to underline the still unsolved aspects of inborn errors of purine metabolism, collecting up-to-date literature on the subject and trying to find, if any, common mechanistic bases.

Different mutations may cause different consequences in the functions of the enzyme objects of this review, such as lower or higher activity, different substrate affinity, impaired regulation, or changes in the ability to interact with other proteins. Thus, it is conceivable that the inborn errors of purine metabolism, which are not easily recognizable without genetic analysis or specific enzyme activity measurement, might be at the basis of unexplained diseases. In other words, the diseases caused by mutations in purine metabolism might be less rare than commonly believed.

## 2. Phosphoribosylpyrophosphate Synthetase

**Figure 2:**
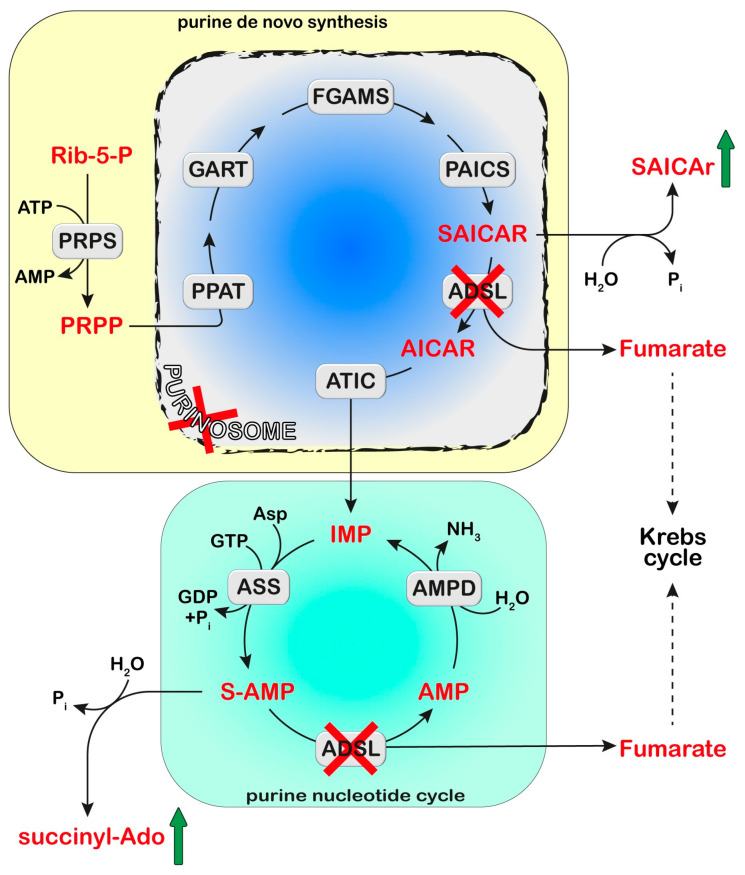
Adenylosuccinate lyase (ADSL) is involved both in the de novo purine synthesis (yellow box) and the purine nucleotide cycle (green box). The deficiency of ADSL causes an accumulation of its substrates, succinyl aminoimidazole carboxamide ribotide (SAICAR) and succinyl-AMP (S-AMP), which are both dephosphorylated and converted to SAICA-riboside (SAICAr) and succinyl-Ado (S-Ado), respectively. The formation of the purinosome complex (blue box) is impaired in cases of ADSL deficiency. Phosphoribosylpyrophosphate (PRPP) is synthesized from ribose-5-phosphate (Rib-5-P) by PRPP synthetase (PRPS). Six enzymes can form the purinosome and catalyze the ten steps required to convert PRPP into IMP: PRPP amidotransferase (PPAT), trifunctional phosphoribosyl glicinamide synthetase/phosphoribosyl glycinamide transformylase/phophoribosyl aminoimidazole synthetase (GART), phosphoribosyl glycinamidine synthase (FGAMS), bifunctional phosphoribosyl aminoimidazole carboxylase/phosphoribosyl aminoimidazole succinocarboxamide synthetase (PAICS), ADSL, and bifunctional 5-aminoimidazole-4-carboxamide ribonucleotide transformylase/IMP cyclohydrolase (ATIC). IMP enters the purine nucleotide cycle composed of adenylosuccinate synthase (ASS), ADSL, and AMP deaminase (AMPD). Asp: Aspartate.

PRPS catalyzes phosphoribosylpyrophosphate (PRPP) synthesis from Mg^2+^-ATP and ribose-5-phosphate, with AMP release (Figure 1 and Figure 2) [15]. This enzyme plays a critical role in nucleotide metabolism, since PRPP is the only phosphoribosyl donor in both de novo and salvage pathways and its concentration regulates the production of the three families of nucleotides (purine, pyrimidine, and pyridine). PRPP is also involved in the synthesis of the two aromatic aminoacids, tryptophan and histidine, in many organisms, but not in humans who lack these synthetic pathways [16]. PRPS activity, regulated by substrates, inhibitors (ADP and GDP), activators (Mg^2+^ and inorganic phosphate), or products, as well as phosphoribosyltransferase activities, are the major factors determining the intracellular level of PRPP [15].

PRPS is widespread in most organisms with numerous isoforms; in humans, three differentially expressed homolog PRPS isoforms have been identified to date. PRPS1 and PRPS2, expressed in all tissues including brain [17], are encoded by *PRPS1* and *PRPS2* genes, respectively, both mapping to the X chromosome [15]. The third isoform, *PRPS1L1*, maps to chromosome 7 and its expression is only detectable in the testes [17].

Numerous scientific reports have shown that mutations in the PRPS genes in different organisms cause various forms of cellular disruption, including neurological diseases and metabolic disorders. In humans, mutations in *PRPS1* lead to a loss- or gain-of-function, both of which, interestingly, cause neurological disorders. A common symptom associated with most PRPS1 mutations is hearing impairment (either nonsyndromic or syndromic hearing loss). Human PRPS1 is a homohexameric enzyme assembled from three tightly interacting dimers containing one catalytic and two allosteric sites per subunit [18]. PRPS has been reported to self-assemble forming filaments in different organisms, including human fibroblasts [19].

### 2.1. PRPS Superactivity

Gain-of-function mutations in *PRPS1* is the better-known pathological alteration of PRPS: a X-linked disorder characterized by UA overproduction with gout. PRPS superactivity appears to be extremely rare, since 33 affected individuals have been described worldwide to date [20]. It may result from defective regulation by purine nucleotides and inorganic phosphate, mostly via the alteration of the allosteric sites [18], catalytic superactivity [21], an increased affinity for ribose-5-phosphate, or combined regulatory and catalytic defects. The accelerated transcription of *PRPS1* has also been found to be responsible for increased activity, with elevated *PRPS1* mRNA [21,22]. Increased PRPP and accelerated purine synthesis account for the metabolic aspects of PRPS superactivity, comprising two phenotypes, both characterized by hyperuricemia and hyperuricuria. The mild phenotype (~75% of affected males) with onset in the second or third decade of life is typically limited to UA crystalluria or urinary stone and gouty arthritis, whereas the severe phenotype (~25% of affected males), which has onset in the first decade of life, displays variable combinations of developmental delay/intellectual disability, sensorineural hearing loss, hypotonia, and ataxia [23]. Women are rarely affected by PRPS1 superactivity due to random X inactivation, a decreased expression of the mutated enzyme, and a higher urate excretion than men. Nevertheless, female patients have also been described, mostly presenting a mild phenotype (only one woman with a severe phenotype has been described so far) [24].

#### 2.1.1. Diagnosis and Treatment

Hyperuricemia and/or hyperuricuria at an early age may be diagnostic features of PRPS superactivity, but differential diagnoses with respect to HPRT deficiency must be considered. In cases of normal HPRT activity, diagnosis can be confirmed by raised PRPS activity and elevated PRPP levels in red cells, often accompanied by low GTP and altered NAD concentrations [20,25]. Molecular gene testing allows for the identification of several *PRPS1* pathogenic variants associated with the severe phenotype. On the contrary, in males presenting with the mild form of the disease, no pathogenic variants were detected in the coding and adjacent sequences of *PRPS1*, and the basis of increased rates of PRPS1 activity is still unknown [20,26]. Hypouricemic drugs such as allopurinol and febuxostat are commonly used to prevent/treat gout, renal failure, and the other metabolic complications of hyperuricemia, but have no known beneficial effect on hearing loss or neurodevelopmental impairment [20]. The neurological manifestations are usually treated by special educators and sensory impairment specialists, but no prevention therapy or effective drug is known. Dietary S-adenosylmethionine (SAM) supplementation, known to cross the blood–brain barrier, may theoretically alleviate some of the neurologic symptoms in individuals with the severe phenotype by providing an oral source of purine nucleotide precursor that is not PRPP dependent. In fact, purine nucleotides (adenylates/guanylates) are high in some cells of these patients, but are low in red blood cells, which rely on purine salvage metabolism, and are believed to be low in the brain, which also relies on purine salvage metabolism. This therapy is experimental and still under investigation [20].

#### 2.1.2. Proposed Mechanistic Basis

No compelling mechanistic explanations for the neurodevelopmental manifestations of PRPS superactivity have been provided. Increased hypoxanthine and xanthine, described as possible toxic metabolites in other pathologies such as Lesch–Nyhan Disease (LND), were reported in the cerebrospinal fluid of a family with PRPS superactivity. Enhanced purine breakdown, leading to both increased oxypurine production and GTP depletion in the central nervous system (CNS), was hypothesized to play a pathogenic role [27]. Detectable amounts of the de novo intermediate derivative 5-amino-4-imidazolecarboxamide, (AICA)-riboside 5′-triphosphate (ZTP), normally absent and suspected to be neurotoxic, has also been reported in the erythrocytes of patients with PRPS superactivity [27]. Altered NAD concentrations have been reported in erythrocytes [27,28], due to decreased synthesis rather than increased degradation [25].

### 2.2. PRPS Deficiency

The loss-of-function mutations in *PRPS1*, identified as missense up to now, may result in destabilizing the ATP binding sites and disturbing the allosteric sites. Likely due to the essential role of PRPS1 in nucleotide metabolism for embryonic development, that supposedly neither PRPS2 nor PRPS1L1 can compensate, the mutant form of PRPS1 retains some activity that allows affected individuals to complete embryonic and fetal development [29]. PRPS deficiency causes rare X-linked diseases, although females exhibiting a severe disease phenotype have been reported [30]. Depending on the level of residual enzyme activity, three medical conditions have been described with features ranging from sensorineural hearing loss (mild enzyme deficiency) to peripheral neuropathy (moderate enzyme deficiency) and to intellectual disability, hypotonia, ataxia, delayed motor development, profound congenital sensorineural hearing impairment, progressive optic atrophy and retinal dystrophy [31], and recurrent infections resulting in early death (severe enzyme deficiency). These phenotypes that have been indicated as distinct entities are now thought to represent a continuum [20]: non-syndromic sensorineural deafness (DFNX1), Charcot–Marie–Tooth type 5 (CMTX5), and Arts syndrome [26]. Progressive hearing loss is an isolated feature in DFNX1 patients, presenting half-normal PRPS1 activity in the erythrocytes and fibroblasts [26]. CMTX5 is associated with mutations in the *PRPS1* gene resulting in halved enzyme activity in patient fibroblasts [32]. Arts syndrome is associated with missense mutations causing remarkable loss of PRPS1 activity (absent in erythrocytes and up to 1/10 of normal in fibroblasts) and is characterized by more severe symptoms [33].

#### 2.2.1. Diagnosis and Treatment

Decreased or completely absent erythrocyte PRPS activity with a normal serum UA concentration can confirm the clinical diagnosis of Arts syndrome, CMTX5, or DFNX1. The treatment of a 3-year old boy with Arts syndrome with SAM replenished the erythrocyte purine nucleotides of adenosine and guanosine, while SAM and nicotinamide riboside co-therapy further improved his clinical phenotype as well as his T-cell survival and function [34].

#### 2.2.2. Proposed Mechanistic Basis

Causative relationships between *PRPS1* mutations and hearing loss were investigated in zebrafish, in which two homologs are present, *Prps1a* and *Prps1b*, with a sequence similarity of 99% with human *PRPS1*. Zebrafish *Prps1a* mutants and *Prps1a*; *Prps1b* double mutants showed similar morphological phenotypes including smaller eyes and reduced hair cell numbers, consistent with the optic atrophy and hearing impairment observed in human patients, and the abnormal development of primary motor neurons, hair cell innervation, and reduced leukocytes that are consistent with the neuropathy, sensorineural hearing impairment, and recurrent infection of human patients with severe phenotype [35,36]. Blocked PRPS filament formation and a disorganized lens fiber actin network was also found in *Prps1a* mutants, highlighting a potential role for PRPS filaments during lens fiber organization [19]. *Drosophila* PRPS shares about 90% protein sequence identity with its human homologs and was also used as a model. By engineering patient-derived missense mutations in *Drosophila*, Delos Santos et al. [29] found some enzyme activity, supporting the hypothesis that enzyme function was not completely eliminated in human patients. The above *Drosophila* model showed macroautophagic/lysosomal defects, not yet investigated in patients, which were suggested to contribute to PRPS-associated neuropathology and could be partially improved by a SAM-enriched diet [29]. De Brouwer et al. [33] hypothesized that the symptoms observed in Arts syndrome may be the result of nucleotide depletion in energy-requiring key tissues, and that the demyelination found in the CNS of one Arts patient at autopsy might be the result of a reduction in pyrimidine nucleotides, essential for membrane and myelin synthesis through their lipid esters.

In conclusion, *PRPS1* may be considered as an example of a human gene in which activating and inactivating mutations cause distinct hereditary disorders with overlapping neurological abnormalities, such as hearing impairment. For some reason, sensory neurons in the auditory system appear highly susceptible to defects of *PRPS1*. Interestingly, the beneficial effect of SAM has been reported in both gain-of-function and loss-of-function disorders [26]. PRPS1-associated genetic disorders might be more prevalent than previously thought.

## 3. Hypoxanthine-Guanine Phosphoribosyltransferase

HPRT catalyzes the salvage of the purine bases, hypoxanthine and guanine, converting them into their respective monophosphate nucleosides (IMP and GMP) by a PRPP-dependent phosphoribosyl transfer reaction (Figure 1). It is the main purine salvage enzyme in humans, cytoplasmic and ubiquitously expressed in human tissues, with the highest activities in the testis and brain and with some significant modifications during fetal life and neonatal development [37,38]. It is encoded by a single structural gene (*HPRT1*) located in the X-chromosome. More than 600 mutations have been described causing various alterations of the aminoacid sequence, the molecular structure, the physical and kinetic properties of the enzyme, or a markedly decreased HPRT expression, leading to different degrees of enzymatic deficiency [37,39,40].

Virtually complete enzymatic deficiency causes LND, a rare and peculiar syndrome, characterized by metabolic and neurological aspects with marked hyperuricemia and hyperuricuria as the hallmarks. Partial HPRT deficiency gives rise to milder phenotypes, all sharing hyperuricemia (LND variants) [40]. Few patients live beyond 40 years, with death occurring due to different causes, including pneumonia and sudden, unexpected death with a respiratory origin. Since HPRT deficiency is inherited as a recessive X-linked trait, males are generally affected, and females may be asymptomatic carriers. Nevertheless, at least five females with complete HPRT deficiency and full LND, and one with partial deficiency, have been reported, due to molecular mechanisms affecting the second allele [41].

Hyperuricemia in HPRT deficiency is due to the marked overproduction of UA through both the catabolism of unrecycled guanine and hypoxanthine and a consistent increase in de novo purine synthesis [37]. Consequently, nephrolithiasis, renal failure, and juvenile gout are common findings in LND and its variants.

LND patients present severe neurological and motor disability, and most of them are confined to a wheelchair. The neurological picture encompasses a spectrum of extrapyramidal signs including dystonia, choreoathetosis, dysarthria, dysphagia, opisthotonos, and occasionally ballismus and pyramidal signs, such as spasticity and hyperreflexia. Behavioral problems are the most striking aspect of the disease: a peculiar compulsive self-injurious behavior, with severe self-mutilation (lip, tongue, or finger biting), and other occasional different means of self-harm, which are not the result of a lack of pain sensation but can be ascribed to an obsessive–compulsive behavior indicated as Lesch–Nyhan Behavior (LNB), which is still under study [37,42]. Psychomotor delay may become evident within 3 to 6 months of age; self-mutilation can appear as soon as teeth are present; cognitive impairment, often described, is difficult to assess due to LNB and is often misdiagnosed [42].

The term “Lesch-Nyhan variants” (LNV) has been introduced to describe a continuous spectrum of neurological involvement present in HPRT-deficient patients correlating with the residual catalytic activity of the enzyme [40], with some degree of cognitive impairment, spasticity, or dystonia, but without the complete syndrome. The finding of surprisingly different phenotypes in members of affected families bearing the same genetic mutation pointed at the involvement of epigenetic mechanisms [43].

### 3.1. Diagnosis and Treatment

Grossly increased UA amounts in plasma and urine is the initial sign, accompanied, in complete HPRT deficiency, by increased hypoxanthine and xanthine. Megaloblastic anaemia unresponsive to folate therapy is common in LND patients and may also raise the suspicion of LND [44]. Hyperuricemia is also present in other enzyme disorders (e.g., PRPS superactivity, glucose 6-phosphate dehydrogenase deficiency, etc.), thus the definitive diagnosis is obtained by either HPRT enzymatic assay in erythrocytes, lymphocytes, or cultured fibroblasts, or molecular genetic testing. The relatively late appearance of the distinguishing LNB can delay diagnosis, and many patients are initially diagnosed with cerebral palsy [37,40].

Inhibitors of XOR, such as allopurinol and febuxostat, effectively lower UA but induce hypoxanthine and xanthine accumulation. The latter may form stones with frequent renal failure [45]; moreover, increased hypoxanthine and xanthine concentrations in LND cerebrospinal fluid have been related to neurological manifestation (reviewed below). Rasburicase, a recombinant urate oxidase converting UA into allantoin, is also sporadically used for the rapid prevention of renal failure. Alternative treatments avoiding hypoxanthine accumulation have been recently proposed, based on recombinant enzyme therapy restoring the uricolytic pathway [46], or on upstream PNP inhibition to slower purine breakdown [47]. Treatment with allopurinol or other hypouricemic drugs has no effect on the neurological or behavioral manifestations of the disease. The lack of a precise understanding of neurological dysfunction in LND has precluded the development of specific and effective therapies. Dopamine replacement therapy in LND patients was proven insufficient to revert neurological symptoms [48]. Current treatments are mainly symptomatic, either by drugs [49] or the chronic deep brain stimulation of the globus pallidus [50].

### 3.2. Proposed Mechanistic Basis

The connection between HPRT deficiency and the neurological syndrome in LND patients has not yet been completely clarified. A number of detailed studies in the last decades have addressed the genetic, metabolic [37,51], cognitive, behavioral [42,43], and anatomical [52] features of the disease and investigated the potentially toxic role of accumulated metabolites or the depletion of essential molecules in patient cells (erythrocytes, lymphoblasts, fibroblasts) in autopsied brain specimens, or in different LND experimental models showing significant differences in cell types, developmental stage, and tissue source [53]. Several metabolic abnormalities were found to accompany HPRT deficiency, besides the grossly increased de novo purine synthesis. Peculiar features were reported in erythrocytes: GTP depletion, increased UDP-glucose, PRPP and NAD concentration [54,55], appreciable levels of ZTP [56], and abnormally increased enzyme activities, namely APRT, IMP dehydrogenase, and cytosolic 5′-nucleotidase II (cN-II) [51]. Decreased NAD, ATP, and GTP concentrations and increased NAD production were measured in LND fibroblasts [57]. NAD metabolism, hypothesized to be involved in LND neurological symptoms, was also altered in the liver, but not in the brain or blood of *Hprt*-knockout (KO) mice [58]. Accumulating lines of evidence indicate dysfunctional dopaminergic (DA) pathways in the brain’s basal ganglia as being mainly responsible for the neurobehavioral dysfunction [48], with the abnormal development of the DA neuronal phenotype, altered brain neurotransmitters (i.e., lower dopamine, serotonin, and 5-hydroxyindolacetate [59,60,61,62]), and the decreased binding to dopamine transporters evidenced by neurochemical and neuroimaging in vivo studies [62]. A marked reduction in early DA neurons, and an abnormal migration of developing DA neurons to the midbrain areas have recently been identified at the embryonic stage in *Hprt*-KO mice; the structural organization of DA target areas (e.g., cortex) was also affected. These findings argue for the early abnormal development of the DA system, rather than a degenerative process [63]. Though no morphological abnormality had been found in postmortem studies of brains from LND patients in the past, advanced imaging methods recently revealed substantial alterations in white matter volume and integrity, with more attenuated declines in LNV [52]. All the above could reflect abnormalities in brain connectivity, pointing at the involvement of pathways beyond the basal ganglia.

Alterations in serotonin and adenosine neurotransmitter systems were also investigated in models of the disease [53,64,65,66] and in LND lymphocytes, revealing variably aberrant expressions in the DRD5 dopamine receptor, the type 2A adenosine receptor, and the type1A serotonin receptor, supporting the hypothesis that neurological manifestations may be related to an imbalance of these neurotransmitters by a coordinated mechanism [66]. Several studies focused on hypoxanthine excess, described as altering adenosine transport in isolated cells [66], and implicated in the morphogenesis impairment and proliferation enhancement of HPRT deficient neuroblastoma cells [67]. Intrastriatal hypoxanthine administration to rats resulted in altered Na+/K+ ATPase activity [68], ATP depletion, mitochondrial dysfunction, and cell death by apoptosis [69]. Altered purine nucleotide concentrations have been postulated as a possible cause of changes in G-protein signaling, also supported by changes in the expression and function of adenylate cyclase C [70]; GTP depletion in HPRT deficiency was indicated to cause morphological changes in DA cell lines [71] and was also hypothesized as affecting tetrahydrobiopterin (BH4) synthesis through GTP cyclohydrolase [72].

Global transcriptomic analyses confirmed that several mechanisms of developmental and cell signaling pathways regulating CNS development are severely affected during neuronal differentiation in HPRT deficient murine and cell models [59,60,73]. In particular, dysregulated WNT signaling, a pivotal player in neural development, has been reported in LND neural stem cells [74], in HPRT-deficient neuroblastoma SH-SY5Y [75], and lung cancer NT2/D1 cell lines [76]. The aberrant expression of genes involved in DA differentiation and DA cell survival, including tyrosine hydroxylase and aromatic L-aminoacid decarboxylase, was also observed [59,60,61,75]. Other relevant transcriptional alterations were involved in energy metabolism [74], cell differentiation and maturation [77], cAMP/PKA signaling [78], cell cycle and division, nucleic acid metabolism [75], and purinergic signaling [79]. Nguyen [80] has suggested that epistasis between mutated *HPRT1* and amyloid precursor protein (*APP*) genes may affect the APP splicing, producing alternative APP fragments that might be responsible for the neurobehavioral syndrome.

Though often not consistent, the reported findings imply that the consequences of HPRT deficiency are far beyond the metabolic ‘‘housekeeping’’ function of this enzyme, and that the pathogenesis of this monogenic neurodevelopmental disease results from combinatorial and multigenic defects. Moreover, the neurological symptoms of LND, likely related to the dysfunction of the DA system in the basal ganglia, possibly extend beyond this area. It is a common opinion that resolutive therapeutic action should be very early, which pushes research to a better understanding of the times and modes of neurological lesion occurrence and progression.

## 4. Adenylosuccinate Lyase (ADSL)

ADSL is a homotetrameric enzyme exhibiting a dual catalytic role: the conversion of succinylaminoimidazolecarboxamide (SAICA)-ribotide (SAICAR) into AICA-ribotide (AICAR) (de novo purine synthesis pathway) and the formation of AMP from adenylosuccinate in the purine nucleotide cycle (Figure 1 and Figure 2). ADSL deficiency is a rare autosomal recessive disorder, first described by Jaeken and Van den Berghe [81], caused by more than 150 different mutations (most of which missense), in the *ADSL* gene [82,83,84,85]. In all cases, the mutations lead to an ADSL enzyme that retains some residual activity, possibly because a complete loss of activity is probably lethal in humans [86]. The clinical presentation includes neurologic symptoms, namely intellectual disability, autism spectrum disorder, microcephaly, and seizures. Three different phenotypes have been reported on the basis of the age of onset and the severity of symptoms: the fatal neonatal form, presenting with hypokinesia, intractable seizures, and respiratory failure; the type I form presenting within the first months of life, characterized by severe psychomotor retardation, microcephaly, seizures, and autistic features; and the type II form, presenting within the first years of life, with moderate or slight psychomotor retardation [86]. Life expectation in ADSL deficiency is variable. The neonatal form may lead to early death, whereas onset in early childhood usually results in a stable course.

### 4.1. Diagnosis and Treatment

ADSL deficiency is usually diagnosed, using HPLC and tandem mass spectrometry, by the detection in extracellular fluids of SAICA-riboside (SAICAr) and succinyladenosine (S-Ado), the dephosphorylated forms of the two substrates of ADSL (Figure 2). Enzyme assay in erythrocyte lysates was not completely reliable due to the tissue heterogeneity of the ADSL defect [87] and residual activity (>2% of normal) could be detected in the lymphocytes or cultured fibroblasts of patients presenting a lethal fetal and early neonatal form of ADSL deficiency [88]. More recently, whole-exome sequencing analysis has become a clinical practice [85,89]. Magnetic resonance imaging can be useful for the clinical diagnosis and for monitoring the progression of the disorder [90]. The severity of the clinical symptoms appears to correlate with the stability of the mutated enzyme and its residual activity [91,92,93]. The ratio of S-Ado/SAICAr, rather than their absolute concentrations, correlates with the severity of the phenotype [94] being less than one in the fatal form, close to one in the severe type I form, and more than one in the moderate type II form. These findings suggest that SAICAr might be the neurotoxic compound, and that S-Ado might counteract its noxious effects, as confirmed in studies conducted in experimental animals and cultured cells [95,96].

No effective treatment is currently available for ADSL deficiency. The therapeutic approach with anticonvulsive drugs is primarily aimed at controlling seizure frequency with minimal side effects [86]. A ketogenic diet has been proposed for the treatment of refractory epilepsy [97,98] and used as a therapeutic tool in several cases of severe ADSL deficiency [89,99]. However, the beneficial effects appeared to be transitory. Treatment with D-ribose was also recommended for the therapy of ADSL deficiency [100]. However, inconsistent effects of the treatment have been reported [89,100,101]. Cultured fibroblasts from ADSL-deficient patients exhibit normal purine nucleotide levels, growth rates, and ATP concentrations, thus suggesting that the symptoms of ADSL deficiency are caused by the accumulation of succinylpurines, rather than by the intracellular deficiency of purine nucleotides [102].

### 4.2. Proposed Mechanistic Basis

ADSL is a component of the purinosome complex, composed of six enzymes catalyzing the ten chemical steps necessary to convert PRPP into IMP [103] (Figure 2). The assembly occurs in the cytosol upon the depletion or increased demand for purines [1]. Using confocal microscopy, Baresova et al. [104] demonstrated that purinosome assembly in the skin fibroblasts of ADSL-deficient patients, cultured in a purine-depleted medium, was significantly impaired and the extent of the enzyme assembly correlated with the severity of the phenotype of ADSL deficiency. The impairment in purinosome assembly reduces metabolite flux through purine de novo synthesis and purine nucleotide recycling in case of a need for purine synthesis; however, the molecular signaling mechanisms that link purinosome formation impairment to clinical outcomes of ADSL deficiency still need to be elucidated.

In the past years, the role of SAICAR in perturbing glucose metabolism has been proposed. In fact, under conditions of limiting glucose, SAICAR has been shown to activate pyruvate kinase M2 (PKM2) [105], the isoform present in cancer, but also in embryonic cells [106]. Therefore, the accumulation of SAICAR, leading to alterations in the activity of PKM2 during development, might contribute to defects in brain maturation in ADSL-deficient subjects. However, how a perturbation in glucose metabolism might reflect in clinical outcomes of ADSL deficiency still needs to be unraveled.

Recently, a step forwards for the understanding of the molecular mechanisms underlying the clinical manifestations of ADSL deficiency has been made using a cell model in which ADSL depletion was obtained by a pool of siRNA in hTERT-immortalized human retinal cells (RPE-1 cells) [107]. The depletion of ADSL caused a p53-dependent proliferation arrest, with no effect on cell death or senescence, and mild DNA damage, both independent of the modulation of purine de novo synthesis. DNA damage could be rescued by the addition of adenosine, thus implicating defects in the purine nucleotide cycle. This observation was sustained in vivo, in the chicken embryo system, where ADSL depletion, obtained by the silencing of the gene, caused the same effect on proliferation and growth, leading to impaired neurogenesis [107]. Dutto et al. [107] also demonstrated that ADSL depletion in RPE-1 cells led to impairments in ciliogenesis. Ciliary defects, leading to microcephaly, were also observed in vivo using a zebrafish model, in which the *Adsl* gene was silenced with antisense morpholino oligonucleotides. These defects were rescued by treatment with methotrexate, which impairs purine de novo synthesis up- and downstream of ADSL [108], and by the inhibition of the enzyme responsible for the formation of SAICAR (Figure 2), but not by nucleoside supplementation. Thus, the authors concluded that the impairment in ciliogenesis was due to the accumulation of SAICAr, and therefore to a damage in the de novo synthesis, rather than to a deficiency in the purine supply [107]. In conclusion, both defects in the purine nucleotide cycle and impaired de novo synthesis may contribute to neurodevelopmental disorders, possibly acting through specific, but unfortunately still unknown, signaling pathways.

## 5. Inosine 5′-Monophosphate Dehydrogenase

Inosine 5′-monophosphate dehydrogenase (IMPDH) catalyzes the first committed step for the synthesis of guanylate compounds. The enzyme is located at a branch point between adenine and guanine nucleotide synthesis and its activity is critical for the regulation of the flux through the two pathways (Figure 1). In humans there are two isoforms of IMPDH that share 84% sequence identity. IMPDH2 is widely expressed and is up-regulated in proliferative cells [109,110], IMPDH1 does not appear to be regulated by proliferative demand and is tissue-specific, being highly expressed in the lung, thymus, and brain [110,111,112]. IMPDH1 is the major isoform in the retina and, in mammals, is expressed as two major splice variants [113,114]. IMPDH has a tetrameric structure, and its binding to ATP or GTP promotes octamer assembly. In vertebrates, IMPDH forms filamentous ultrastructures, whose conformation determines the activation or partial/total inactivation of the enzyme [115]. For a detailed description of the filament architecture and the regulation of the assembly, the reader is referred to recent and extensive studies. Briefly, the binding of ATP favors the formation of a more extended, more active conformation, while the binding of GTP generally leads to a more compressed, less active form [114,116].

Missense mutations lead to disease in humans. Mutations in the *IMPDH1* gene cause autosomal dominant retinal degeneration, namely retinitis pigmentosa type 10 (RP10) or Leber congenital amauriosis type 11 [116,117,118,119,120], while mutations in the *IMPDH2* gene have been only recently discovered and lead to severe juvenile neuropathies [121,122]. RP10 is characterized in most patients by early onset and the rapid progression of ocular symptoms, beginning with night blindness in childhood, followed by a loss of the peripheral visual field and eventually by complete blindness. IMPDH2 deficiency is characterized by dystonia and tremor, and its onset has been evaluated between 9 and 20 years of age.

### 5.1. Diagnosis and Treatment

Genetic analyses carried out in families from various geographic origins revealed the presence of mutations in the *IMPDH1* gene [118,119,120,123]. Whole-exome sequence analysis was employed for the recent identification of *IMPDH2* variants in dystonia-affected individuals [121] and in a Finnish family affected by juvenile-onset dystonia-tremor disorder [122]. The RNAi-mediated ablation of IMPDH1 transcripts in a murine model of RP10 appears to be promising [124], and gene therapies are under investigation for the treatment of certain types of Leber congenital amauriosis [125,126]. However, to our knowledge, no specific treatment for IMPDH1-associated RP and IMPDH2-associated neuropathy has been reported.

### 5.2. Proposed Mechanistic Basis

Despite the wide tissue distribution [110], the retina appears to be the only tissue affected by IMPDH1 mutations. This might be due to a specific purine requirement in the retina [127], and cyclic guanosine monophosphate (cGMP) demand for the phototransduction cascade [128].

So far, 12 IMPDH1-linked RP mutations have been described and none of them appeared to alter the specific activity of the enzyme [115,116,129,130]. Six of these IMPDH1 mutations disrupt the feedback inhibition by GTP [114,115,116]. Therefore, it has been proposed that the effect of these IMPDH1 mutations on photoreceptors might be due to the dysregulation of purine synthesis, such as the excessive production of cGMP, which is likely to be a major factor in retinal degeneration [131]. Other mechanisms need to be taken into account for the mutations that do not interfere with IMPDH1 regulation. In this regard, IMPDH1 exerts several functions in the retina, which are not strictly related to its catalytic activity, such as binding single stranded DNA [130], and the association with polyribosomes translating rhodopsin [132]. In addition, three phosphorylation sites were recently described in retinal IMPDH1 [133]. It has been demonstrated that a few mutations reduce phosphorylation [133] and disrupt the association with polyribosomes [132], thus possibly resulting in retina degeneration.

Zech et al. [121] first described mutations in the *IMPDH2* gene in a cohort of paediatric individuals presenting with dystonia. Further insight on the disorder was obtained in a study performed in a Finnish family, where a heterozygous truncating variant in the *IMPDH2* gene was identified [122]. The study, performed on induced pluripotent stem cell (iPSC) lines generated from dermal fibroblasts, revealed an almost complete IMPDH2 protein depletion in the patients’ neural lineage. Although further investigations are needed, Kuukasjarvi et al. [122] hypothesized that IMPDH2 deficiency may impair guanylate and eventually dopamine synthesis. In fact, the conversion of GTP into BH4, necessary for dopamine biosynthesis, is often affected in genetic dystonia [134]. Indeed, mutations in the enzymes necessary for the synthesis of BH4, such as HPRT or GTP cyclohydrolase, are associated with a dystonic phenotype [62,135], though BH4 limitation did seem to be responsible for the dopamine loss in LND patients and in animal models [72]. A further similarity with HPRT deficiency was the observed guanine nucleotide depletion induced by an inhibition of IMPDH with low levels of mycophenolic acid in human neuroblastoma DA line LAN5, which developed less extensive neurite outgrowth and branching, similar to that observed in cultured HPRT-deficient DA neurons [71].

## 6. 5′-Nucleotidases

The family of 5′-nucleotidases is composed of one ectosolic (eN) and six cytosolic (cN) members. The enzymes catalyze the hydrolysis in the 5′-position of both ribo-and deoxyribo-nucleoside monophosphates, with the formation of an inorganic phosphate and the corresponding purine or pyrimidine nucleoside (Figure 1). Ectosolic 5′-nucleotidase was the first enzyme of the family to be purified and studied [136]. The protein, which is strongly inhibited by ATP [136], is linked to the cell membrane through a glycosyl phosphatidylinositol anchor and its role is the hydrolysis of both purine and pyrimidine extracellular nucleoside monophosphates, generating nucleosides, that may act in the purinergic signaling, or may enter the cell through equilibrative or concentrative transporters. Inside the cell, nucleosides may be salvaged or further catabolized [137,138]. Among cytosolic nucleotidases, cN-IA is located mainly in skeletal muscle and the heart [139], and preferentially hydrolyzes AMP, but also pyrimidine monophosphates. CN-IB is expressed ubiquitously, is highly homologous to cN-IA, and its substrate is AMP. CN-II is an ubiquitous enzyme, its expression is higher in proliferating tissues and low in muscle [140], and its best substrates are IMP and GMP, but also AMP may be efficiently dephosphorylated, despite the high Km for this substrate [140]. CN-III is expressed in erythrocytes and is believed to regulate the degradation of pyrimidine nucleotides. Finally, cytosolic deoxynucleotidase (dNT-1) and mitochondrial deoxynucleotidase (mdN) preferentially act on pyrimidine deoxynucleotides. All the above-described dephosphorylating activities are often contemporaneously present in the same organ and, since they show a partially overlapping substrate specificity, it is not easy to identify the enzyme responsible for nucleotide degradation in different physiological situations. Nevertheless, we may conclude that cN-I and cN-II are the major nucleotidases involved in the catabolism and recycling of purine compounds inside the cells.

### 6.1. Ectosolic 5′-Nucleotidase

In 1997, Page et al. [141] described a syndrome later named nucleotidase-associated pervasive developmental disorder (NAPDD) [142]. The gene involved in this disorder was not identified, but in fibroblasts of one NAPDD patient, a hyperactivity of eN was demonstrated [143]. The syndrome was characterized by extreme hyperactivity and impulsiveness, with a short attention span, poor social interaction, and aggressiveness. The disease is extremely rare; the patients are delayed in language and some are non-vocal. All patients show neurological symptoms such as seizures, ataxia, an awkward gait, and impaired fine motor control. As observed in other purine-linked pathologies, patients often show infections of the sinuses and middle ear [141]. The patients show low uricosuria, low immunoglobulins, and sometimes decreased T-cell function. The relationship between the enzyme hyperactivity and the symptoms has not yet been elucidated.

**Figure 3:**
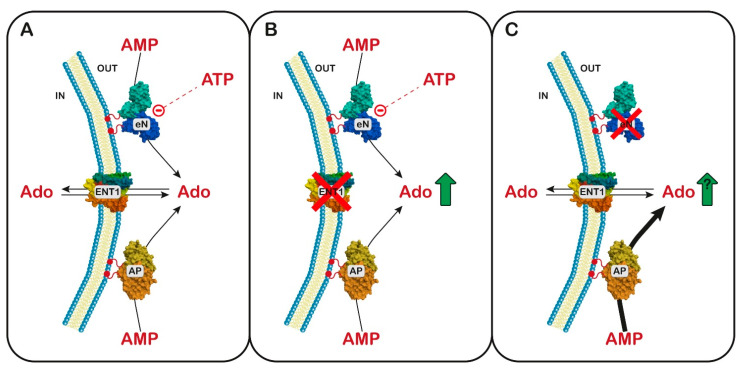
Hypothesis of a common mechanism between equilibrative nucleoside transporter 1 (ENT1) and ectosolic 5′-nucleotidase (eN) deficiency. Panel (**A**) Ado, formed by eN, whose activity depends on the oscillatory levels of ATP, and nonspecific alkaline phosphatase (AP), can cross the cell membrane through ENT1: Panel (**B**) ENT1 deficiency is accompanied by an increase in extracellular Ado. Panel (**C**) In case of eN deficiency, the level of Ado is no more subjected to the oscillatory concentration of ATP, and the activity of AP is up-regulated, thus possibly leading to a paradoxical increase in extracellular Ado.

A mutation causing the loss of function of eN has also been described and is associated with a very rare adult-onset vascular disease, with calcification of joints and arteries [144]. Mouse animal models, which were prepared to better understand the molecular mechanism linking the enzyme deficiency to the symptoms of the disease, did not completely reproduce the human phenotype [145]. In fibroblasts of patients affected by eN deficiency, a stimulation of protein kinase B (PKB or AKT) was reported, with activation of the Forkhead box protein O1, which promoted the hyperexpression of membrane-bound tissue-nonspecific alkaline phosphatase, which, besides phosphate esters, can efficiently hydrolyze pyrophosphate. Since pyrophosphate is an endogenous mineralization inhibitor, its removal has been reported to favor ectopic calcification [146]. Curiously, the same symptomatology was reported in three siblings of European origin and was associated with a mutation of the adenosine equilibrative transporter ENT1, which completely destroyed its function [147]. Furthermore, ENT1 null mice showed the same calcifications of the bone joints [148]. The dysfunctions of ENT1 are associated with increased extracellular adenosine, which is an important promoter of osteogenic differentiation in mesenchymal stem cells through an interaction with A1 and A2B receptors [149]. Since eN loss of function is expected to lower extracellular adenosine concentration, a simple model to explain these contradictory lines of evidence has not been proposed. In this regard, it is worth mentioning that, in the case of eN loss of function, a concomitant increase in tissue-nonspecific alkaline phosphatase has been measured [146]. This enzyme can efficiently hydrolyze AMP, releasing adenosine, and is not subjected to the inhibitory regulation exerted on eN by low concentration of ATP [150]. Therefore, a paradoxical increase in extracellular Ado in cases of eN loss of function can be hypothesized, which could explain why a deficiency in ENTI and eN are characterized by the same clinical manifestation (Figure 3).

#### Diagnosis and Treatment

The diagnosis of eN gain- or loss-of-function requires the measurement of 5′-nucleotidase activity in cultured fibroblasts and the identification of the involved isoenzyme [143]. The therapy that was successful in relieving the symptoms of NAPDD was the administration of uridine or UMP plus CMP or ribose [141]. It was hypothesized that uridine may help in correcting the slight decrease in uridylate measured in these patients [141], but it is worth mentioning that uridine is transported by the same system active on adenosine [151] and therefore an excess of the pyrimidine nucleoside might interfere with adenosine trafficking across the cell membranes and thus with its function in neurodevelopment and inflammation.

### 6.2. Cytosolic 5′-Nucleotidase I

It has been demonstrated that cN-IA interacts with contractile elements in cardiac muscle [152]. The enzyme is responsible for AMP hydrolysis and adenosine production in ischemic conditions, since it is activated by ADP. This activity was demonstrated in the rat cardiomyocytes cell line H9c2 and neonatal rat cardiomyocytes under conditions of ATP breakdown, even though the Km for AMP is in the mM range [139,153,154]. Although cN-IA has been studied mostly for its role in intracellular adenosine formation in the heart, the low Km values for deoxyribo pyrimidine nucleoside monophosphates suggest that the enzyme may also be important in regulating pyrimidine deoxynucleotide pools in the tissues where it is expressed [155]. The enzyme expression has not been associated with specific pathologies, but anti cN-IA antibodies were also found in inclusion body myositis, an acquired, late-onset inflammatory myopathy, with both inflammatory and degenerative pathogeneses. Anti-cN-IA antibodies were found in other autoimmune diseases, such as Stevens–Johnson syndrome, systemic lupus erythematosus, juvenile dermatomyositis, and others, and even in healthy controls [156,157]. The presence of antibodies was associated with a decrease in muscular cN-IA activity and also with some mitochondrial dysfunction. It was hypothesized that the decrease in cN-IA activity caused the AMP accumulation and the activation of the AMP-activated protein kinase (AMPK), which in turn inhibited the mechanistic target of rapamycin (mTOR). The signaling caused an inhibition of protein synthesis and mitochondrial dysfunction, contributing to muscle weakness and degeneration [158].

### 6.3. Cytosolic 5′-Nucleotidase II

The cN-II physiological role is expected to be the hydrolysis of relatively high concentrations of IMP and AMP, thus controlling purine intracellular concentrations and the regulatory functions of AMP and adenosine. Nonetheless, how cN-II activity impacts on these functions has not yet been completely clarified [140]. Since 2009, nine mutations in the gene encoding for cN-II (*NT5C2*) have been associated with a diagnosis of hereditary spastic paraplegia 45 (HSP45) [159,160]. HSP is a rare group of neurodegenerative disorders with various genetic origins and clinical presentations, characterized by a progressive lower limb spasticity and weakness that results from a loss of corticospinal motor tract function [161]. In several cases, the described mutations strongly impact the protein, generating shorter transcripts [162]. Therefore, it is reasonable to infer that HSP45 is associated with a complete lack of functional cN-II. Recently, a large consanguineous Saudi family was reported, co-segregating a novel homozygous splice site donor alteration in *NT5C2* with a phenotype of spastic diplegic cerebral palsy, developmental delay, and microcephaly [163]. Further investigation is necessary to understand the link between the cN-II mutations and the described diseases, but the observation clearly indicates the relevance of cN-II for neurodevelopment. It is worth underlining that cN-II might be important, not only for its enzyme activity, but also for its ability to interact with other proteins, such as the ICE protease-activating factor, involved in inflammation [164], or with other proteins as indicated in protein–protein interaction databases (BioGRID and IntAct-EMBL-EBI). Other suggestions on the impact of cN-II expression and function in physiological and pathological conditions came from several genome wide association studies (GWAS), demonstrating a strong association between mutation in the locus containing the gene coding for cN-II and a plethora of neurological disorders including schizophrenia and autism [160]. In several cases, the frequency of single-nucleotide polymorphism (SNP) within, or nearby, the gene sequence was measured [165,166]. A specific SNP, inside the gene sequence, was first identified and associated with schizophrenia in a meta-analysis from 17 independent studies and confirmed within a validation set [167], and later in a South Chinese Han population [168]. The same SNP was found in association with bipolar disorders in a Latino cohort [169]. Genetic variants in the cN-II locus have also been associated with high blood pressure [170,171] and body weight [172,173]. Unfortunately, it is presently unknown whether the described variants affect the expression, activity, and/or binding capacity of cN-II. However, some genetic variants, located in the vicinity of the coding sequence of cN-II, play a role in the regulation of the transcription of the enzyme, as demonstrated for schizophrenia risk variants affecting the miR-206 function in the regulation of cN-II expression [174].

#### 6.3.1. Diagnosis and Treatment

As reported above, so far, mutations in *NT5C2* were associated with various diseases, whose pathogenesis may also be ascribed to other factors. Therefore, a specific diagnosis relies only on genetic analysis. As far as we know, no attempt to propose a therapy based on cN-II involvement has been made so far.

#### 6.3.2. Proposed Mechanistic Basis

To better understand the impact of cN-II activity on nucleotide metabolism and pathological manifestations, several cellular models hyperexpressing or silenced for cN-II were designed, but inconsistent results were often obtained. Particularly, the relevance of cN-II expression on AMP accumulation is questionable, because different authors report contradictory results. In fact, cN-II silencing was accompanied by AMPK activation in myotubes [175]. However, other authors report that the manipulation of cN-I is more efficient in regulating AMP intracellular concentrations and AMPK activity [176]. Recently, cN-II silencing in tumor cells of epithelial origin indicated that a total absence of cN-II expression was followed by a decrease in proliferation and motility, and the activation of AMPK and p53 [177]. So far, it has been reported that cN-II was silenced in zebrafish [178], in *Drosophila* [179], and in mouse [180]. CN-II silencing in zebrafish confirmed that the enzyme expression is involved in blood pressure regulation. In fact, larvae knockout for cN-II showed higher blood flow, increased arterial pulse, and elevated linear velocity. CN-II was also silenced in *Drosophila* and in human neural progenitor cells by Duarte et al. [179], in an attempt to better understand the function of cN-II expression in neuropsychiatric disorders, as outlined by GWAS [160,165,168]. The results demonstrated that the *Drosophila* model possesses a cN-II homolog very similar to the human enzyme and that its knockdown was associated with abnormal climbing behavior, supporting a role for cN-II in the development of motility, and in diseases associated with motor symptoms. The silencing of cN-II in human neural progenitor cells demonstrated an activation of AMPK and a consequent decreased capacity to synthesize proteins [179]. A genetic deletion of cN-II was obtained in mice by Kviklyte et al. [180]. Their results clearly indicated that no particular problems were caused by silencing in muscle cells, and no AMPK activation could be demonstrated. The animals were apparently in good health and did not exhibit particularly striking behavioral or metabolic phenotypes. However, once fed with a high fat diet, they displayed lower weight and lower fat gain with respect to control mice. The rate of lipolysis was enhanced in silenced mice and insulin sensitivity was higher with respect to controls [181]. The causal relationship between this observation and cN-II has not yet been clarified, but the result in this model is in line with studies demonstrating an association between SNP in the locus of the cN-II gene and body weight [160,182].

## 7. Adenosine Monophosphate Deaminase

Adenosine monophosphate deaminase (AMPD) converts AMP to IMP (Figure 1 and Figure 2). In humans, there are different isoforms of AMPD: AMPD1, highly expressed in skeletal muscle, AMPD2 which is more widely expressed, and AMPD3, expressed mainly in erythrocytes, but also in smooth muscle and type I (slow twitch/oxidative) muscle fibers [183,184]. Altered isoforms 1 and 2, but not 3, are known to result in pathological states.

### 7.1. AMPD1 Deficiency

A lack of AMPD1, the muscle-specific isoform of AMPD (myoadenylate deaminase deficiency), can cause a metabolic myopathy, with exercise-induced muscle symptoms such as early fatigue, cramps, and/or myalgia [185,186,187]. More recently, hypersomnia has also been described [188]. Although 1–2% of the Caucasian population carries one of the mutations that cause myoadenylate deaminase deficiency, few carriers show symptoms [183] and the clinical relevance of AMPD1 deficiency has been questioned [189].

#### 7.1.1. Diagnosis and Treatment

The diagnosis involves histochemical staining or a biochemical analysis of a muscle biopsy demonstrating loss of muscle AMPD activity, or an identification of the mutation. The oral administration of approximately 2 g of D-ribose for 4 days was reported to have a beneficial effect on one patient [190], but improvement has not been found in another study in which a similar dose was used [191]. Other authors have found improvement of muscle functionality using the repetitive administration of higher doses, before and during exercise [192,193]. It is unclear whether the different effect may be due to the amount of a given ribose, or to the timing of the dosing. The protective effects of ribose, if any, might be due to its action as an energy source and to the increase in the de novo synthesis of purine nucleotides [192].

#### 7.1.2. Proposed Mechanistic Basis

AMPD1 is necessary in maintaining the right balance of adenylate pool necessary for a high energy charge or a high energy yield of ATP hydrolysis. The lack of overt effects in human and animal models suggest that AMPD1 deficiency probably requires the loss of compensation mechanisms or the existence of other impairments in muscle metabolism to result in the manifestation of muscular symptoms. The discrepant reports on the effect of reduced AMPD1 activity on physical performance may be the result of differences in exercise, in exercise intensity, or the muscle fiber type involved [194,195,196,197,198]. Based on results from *Ampd1* knockout mice, Hafen et al. [198] have suggested that the consequences of AMPD1 deficiency in humans could not be apparent in individuals with a high percentage of slow-twitch fibers (with low AMPD1 expression) or in those with a greater mixed-fiber composition, such as an untrained population [198]. Hypersomnia could be due to the modulatory effect in the sleep circuits of adenosine, which is able to cross the blood–brain barrier [188] and may accumulate in AMPD1-deficient individuals as a result of cNs activity on AMP. The increase in adenosine might also be the cause of the protective effect of AMPD1 deficiency on coronary arterial disease and heart failure [199,200,201].

### 7.2. AMPD2 Deficiency

Pontocerebellar hypoplasia type 9 is caused by biallelic variants in the gene coding AMPD2. The patients also show a combination of postnatal microcephaly, hypoplastic or absent corpus callosum, and severe intellectual disability [202,203,204]. In addition, an AMPD2 variant has been reported in two patients with HSP63 [205]. It is possible that HSP63 could be the result of the presence of some intact isoforms of AMPD2, while more severe and earlier-onset pontocerebellar hypoplasia could derive from a broader absence of the enzyme [202].

#### 7.2.1. Diagnosis and Treatment

Patients demonstrate severe neurodevelopmental delay with early onset and typical magnetic resonance imaging [206]. The diagnosis is obtained by sequencing the AMPD2 gene. Enzymatic activity assays and an enzyme-linked immunosorbent assay can also be utilized to confirm loss or reduction in AMPD2 using lysed fibroblasts or peripheral blood mononuclear cells [207].

#### 7.2.2. Proposed Mechanistic Basis

Studies using fibroblasts of patients have shown that AMPD2 mutant cells grow normally and have normal nucleotide levels but show adenosine-induced cell death [208]. Moreover, cytotoxicity does not depend on the activation of receptor-dependent purinergic signaling. Patient cells show the adenosine-dependent accumulation of ATP and the depletion of guanine nucleotides [208]. AMPD2 deficiency results in a defective GTP-dependent initiation of protein translation, which can be rescued by the administration of purine precursors such as AICAr, but not by the AMPK activator metformin. This result excluded AMPK activation as the protective mechanism. Although *Ampd2* knockout mice did not show a neurodegenerative phenotype, the double knockout *Ampd1/Ampd2* did. The difference in brain vulnerability in mice and humans could be explained by the different expressions of AMPD isoforms. In mice, AMPD1 and AMPD2 are coexpressed in the brain and contribute equally to brain AMPD activity. Interestingly, *AMPD2* and *AMPD3* are coexpressed in the developing human cerebral cortex, but AMPD2 is predominant in the cerebellum [208]. The potential of AICAr as a therapeutic treatment remains to be studied. However, AICAr has been demonstrated to be toxic for neuroblastoma cells (SH-SY5Y) and immortalized hippocampal cells [51], and to inhibit axon growth [209].

## 8. Adenosine Kinase

Adenosine kinase (ADK) catalyzes the transfer of the γ-phosphate from ATP to adenosine, leading to the formation of AMP (Figure 1), and regulates both extracellular adenosine and intracellular adenine nucleotide levels. Human ADK consists of two alternatively spliced forms with distinct cellular and subcellular localization and functions. The overexpression of shorter, cytoplasmatic ADK in the brain resulted in spontaneous seizures and increased brain injury after ischemic stroke but the overexpression of the longer, nuclear ADK in dorsal forebrain neurons attenuated neural stem cell proliferation [210]. In addition, nuclear ADK might have a more prominent role in epigenetic mechanisms requiring transmethylation reactions than cytoplasmic ADK [211].

ADK deficiency is a rare inborn error of methionine and adenosine metabolism, first clinically described in 2011 [212]. So far, less than 30 patients have been reported [210,213,214,215] as showing liver dysfunction, delayed psychomotor development, mild dysmorphic features, and neurological features including generalized hypotonia and epilepsy. Vascular abnormalities have also been recently reported [216].

### 8.1. Diagnosis and Treatment

The patients present persistent hypermethioninemia with increased levels of SAM and SAH, while homocysteine levels are normal. The final diagnosis is usually confirmed by genetic studies, including *ADK* gene sequencing or whole exome sequencing. There is no treatment for this disease, but methionine restriction has resulted in improvement in some patients [213,215,217].

### 8.2. Proposed Mechanistic Basis

The mechanism in ADK deficiency is unclear; however, it is likely to involve a reduction in energy metabolites (ATP), the inhibition of transmethylation reactions, and an alteration of adenosine signaling via adenosine receptors.

**Figure 4:**
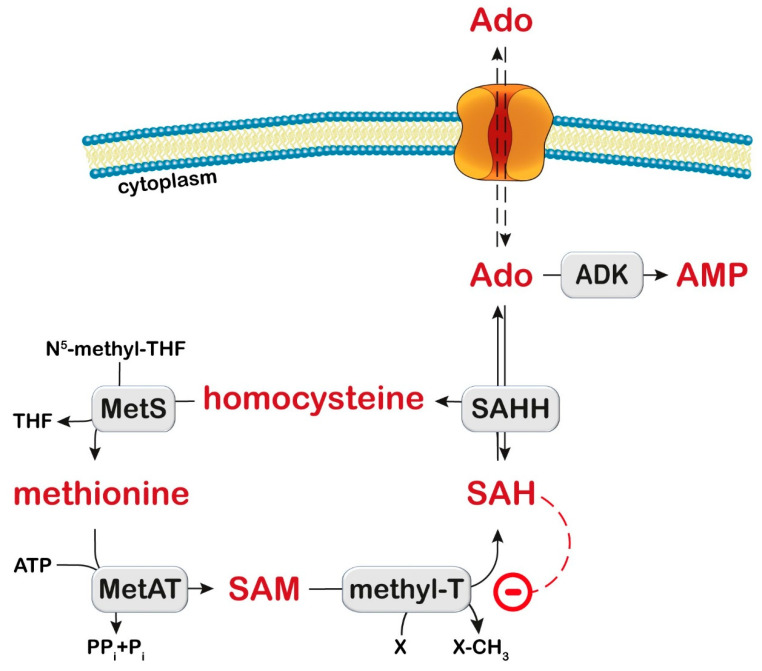
Transmethylation pathway. S-Adenosylhomocysteine hydrolase (SAHH) catalyzes the reversible reaction which converts S-adenosylhomocysteine (SAH) to homocysteine and adenosine (Ado). Ado can be phosphorylated to AMP by adenosine kinase (ADK). Methionine synthase (MetS) converts homocysteine into methionine. Methionine adenosyl transferase (MetAT) catalyzes the formation of S-adenosylmethionine (SAM). SAM is the donor of the methyl group in the transmethylation reactions catalyzed by methyltransferases (MethylT). X: acceptor of the methyl group. SAH is an inhibitor of MethylT (dashed line). THF: tetrahydrofolate; PPi: inorganic pyrophosphate; Pi: inorganic phosphate.

The methionine cycle comprises a series of enzymatic conversions, including adenosylation of methionine by methionine adenosyl transferase to SAM, which is a donor of the methyl group (Figure 4). Then, methyltransferases catalyze the addition of the methyl group to many substrates (proteins, DNA, mRNA, lipids, cathecols, and many other compounds) generating SAH, which is hydrolyzed by SAH hydrolase (SAHH) to generate adenosine and homocysteine. Homocysteine is then either remethylated to methionine or transsulfurated via cystathionine to cysteine. ADK deficiency leads to a disruption of the methionine cycle by adenosine accumulation resulting in a reversal of the SAHH reaction, SAH increase, and methyltransferase inhibition [218] (Figure 4). In the liver of rats fed with methionine, an increase in methionine, SAH, SAM, oxidative damage to mitochondrial DNA, and a decrease in complex IV levels were observed, with reduced ATP production [219]. The ATP depletion may impair mitochondrial fatty acid oxidation and contribute to steatosis. Interestingly, methionine restriction was able to decrease oxidative damage to mitochondrial DNA, protein oxidation, and lipoxidation in rats [220].

Homozygous *Adk (-/-)* mice developed normally during embryogenesis but within 4 days after birth they displayed microvesicular hepatic steatosis and died within 14 days with fatty liver disease [221]. Therefore, this model demonstrated the liver dysfunction, but it was not possible to study the neurological manifestations. Decreased concentrations of adenine nucleotides were measured in liver homogenates, suggesting energy deficiency as a possible mechanism for liver steatosis [221].

The mechanism underlying neurological manifestations was studied when mice with a brain-wide deletion of *Adk* were available. These mice developed spontaneous seizures and profound deficits in hippocampus-dependent learning and memory (fear conditioning paradigm), but not in working memory [222]. These manifestations were unexpected since adenosine has neuroprotective and anticonvulsive effects [223], but reflected the phenotype of human patients. Astrocytic ADK expression is known to increase in both animal models and in the hippocampus of epileptic patients [211]. Indeed, seizures in murine models involve the alteration of adenosine homeostasis (increased ADK and reduced adenosine), and seizure susceptibility is reduced by DNA methyltransferase inhibitors [211]. The solution of this conundrum might be that ADK deficiency in the brain triggers adaptation processes that lead to dysregulated synaptic plasticity. Indeed, long-term potentiation (considered a cellular model of memory) was enhanced in the hippocampus of *Adk-*deficient mice, and this was dependent on adenosine receptor 2A (A2AR) and brain-derived neurotrophic factor (BDNF) signaling [222]. The increase in adenosine levels due to ADK deficiency allows for the stimulation of A2ARs, which are less abundant and require more adenosine than A1AR. A2AR blockade and a BDNF receptor antagonist reduced seizures. Mutant mice, in which both brain *Adk* and the gene coding forA2AR have been deleted, showed improvement in the cognitive deficits compared with the brain of *Adk*-deficient mice [222].

## 9. Adenosine Deaminase

ADA catalyzes the deamination of adenosine and deoxyadenosine to inosine and deoxyinosine, respectively (Figure 1). Two isoenzymes are present in humans: ADA1, which is found in most tissues, and ADA2, which is found in monocytes and macrophages. Both ADA1 and ADA2 play a crucial role in the differentiation and function of immune cells [224]. ADA1 can directly interact with dipeptidyl peptidase-4 (DPP4), also named CD26, and can also exist as a complex formed by two ADA1 subunits bound to two subunits of DPP4/CD26 on the cell surface, facilitating the extracellular breakdown of adenosine. ADA1 can also interact with AR type 1 and 2, modulating receptor functionality [224]. The formation of trimeric complexes DPP4-ADA1-A2AR have been also demonstrated and a role has been proposed for ADA1 in communication between cells expressing DPP4/CD26 (such as T-cells) and those expressing adenosine receptors (such as dendritic cells and neurons) [225]. Adenosine induces immunosuppression via the activation of A2AR and the accumulation of intracellular cAMP, thus preventing edema and excessive inflammation [226]. ADA1 bound to DPP4/CD26 at the cytoplasmic membrane reduces potentially harmful extracellular adenosine levels and prevents the persistent activation of adenosine receptors.

ADA2 belongs to a family of proteins first described as growth factors in insects [227] and is coded by the *ADA2* gene [224,228,229]. Human ADA2 induces the differentiation of monocytes into macrophages and stimulates the proliferation of T helper cells and macrophages [230]. ADA2 has 100-fold lower Km than ADA1, an acidic optimum pH for activity, and the structure of its catalytic domain exhibits a different arrangement of the substrate-binding pocket that may determine the selective inhibition of ADA1 but not ADA2 by erythro-9-(2-hydroxy-3-nonyl)adenine [231]. ADA2 is a minor component of total ADA activity in human tissues, but it is secreted by activated monocytes and it is the predominant activity in human plasma [232].

### 9.1. ADA1 Deficiency

ADA1 deficiency is the second most common cause of severe combined immunodeficiency (SCID), accounting for 15% of all cases. ADA-deficient SCID is an inherited autosomal recessive disease caused by the complete or partial loss of ADA1 activity. Over 70 causative mutations in the *ADA* gene have been identified that give varying levels of ADA1 activity in the host [233]. In addition to severe and recurrent infections, with lymphocytopenia and the absence of both humoral and cellular immune function, patients present neurodevelopmental deficits, behavioural disorders, sensorial deafness, and skeletal and hepatic abnormalities [210,233].

#### 9.1.1. Diagnosis and Treatment

The diagnosis of ADA1-deficiency is established by biochemical and molecular genetic testing. Absent or greatly reduced ADA1 activity (<1% of normal) and the marked elevation of dATP or total deoxyadenosine nucleotides (dAMP, dADP, and dATP) are found in erythrocytes [233]. Treatment consists of hematopoietic stem cell transplantation, enzyme replacement therapy with polyethylene glycol-modified ADA1 (PEG-ADA1), or gene therapy by an infusion of marrow cells transduced with an ADA-containing vector [234,235].

#### 9.1.2. Proposed Mechanistic Basis

When ADA1 activity is absent, both the extracellular and intracellular deoxyadenosine amount increases. Inside the cells, it can be converted to dAMP and then to dATP. Deoxyadenosine and dATP are cytotoxic in lymphocytes. Increased levels of dATP inhibit ribonucleotide reductase and terminal deoxynucleotidyl transferase, causing an imbalance in other dNTP [236] and impairing DNA synthesis and repair, inducing apoptosis of developing thymocytes [210,233]. Deoxyadenosine inactivates SAHH, leading to the accumulation of SAH, and inhibits the transmethylation reactions (Figure 4) which are required for lymphocyte activation. The accumulation of adenosine and deoxyadenosine might contribute to the alterations in the nervous system. Although bone marrow transplant or replacement therapy improve the immunological and metabolic aspects of the disease, they are inefficient regarding the neurological deficits [237,238]. Experiments performed in *Ada−/−*mice treated with PEG-ADA1 [239] have demonstrated that, although these mice have normal sensory motor development, their brain size and pain sensitivity are reduced and anxiety is increased. In addition, A2AR levels in *Ada−/−* mice are lower than in those of the control brains, even when treated with PEG-ADA1. An earlier treatment could correct some neurological deficits, such as hearing defects in *Ada−/−*mice [240]. ADA enzyme therapy in these mice normalized cochlear adenosine levels and prevented demyelination. Moreover, treatment with an A2BR-antagonist improved hearing loss and myelin compaction [241]. These observations suggest that ADA1 deficiency impairs adenosine signaling but the involvement of other mechanisms, such as epigenetic alterations, cannot be ruled out.

### 9.2. ADA2 Deficiency

Deficiency of ADA2 is an autosomal recessive disorder, caused by loss-of-function mutations in the ADA2 gene leading to a reduction in enzyme activity [242,243]. The disease presents in early childhood with symptoms including autoinflammatory, vasculopathic, hematologic, and immune system dysfunction [244]. ADA2 deficiency, like ADA1-deficient SCID, also has a range of neurological manifestations. Ischemic strokes are a common feature [243], with imaging of the brain showing lacunar lesions in the brain stem [245], the effects of which can accumulate over time to induce more severe neurological symptoms such as dysarthria, ataxia, palsy, and cognitive impairment. Other neurological manifestations included intracerebral haemorrhaging [242,246,247], central and peripheral neuropathy [248], and aneurysms [229]. Neuroimaging has also more recently demonstrated that patients develop cerebral microbleeds and inflammatory perivascular tissue in the basal and prepontine cisterns [249]. The severity of the manifestations appears to depend on the residual enzymatic activity. It has been proposed that mutations with at least 3% residual enzymatic activity of ADA2 are associated with vasculitis, whereas pure red cell aplasia and bone marrow failure result from mutations with minimal residual activity or a complete loss of function [248].

#### 9.2.1. Diagnosis and Treatment

The diagnosis of ADA2 deficiency is established through the identification of biallelic loss-of function ADA2 pathogenic variants and/or low (<5% of normal) or undetectable ADA2 catalytic activity in plasma or serum [228]. Tumor necrosis factor α (TNFα) inhibition resolves vasculopathy, but not cytopenia [250]. Hematopoietic cell transplantation appears to be an effective treatment, reversing refractory cytopenia together with vasculopathy and immunodeficiency [250].

#### 9.2.2. Proposed Mechanistic Basis

**Figure 5:**
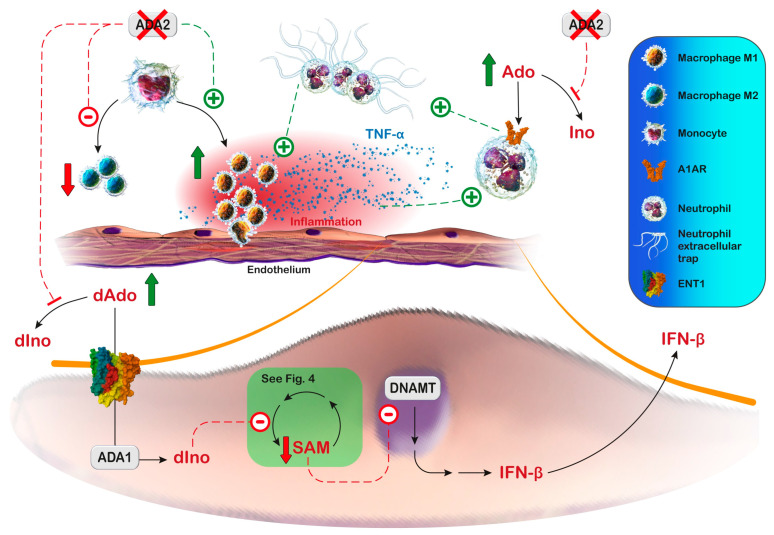
Proposed mechanisms of vascular inflammation in deficiency of ADA2. In the absence of ADA2 activity, there is a decrease in macrophages M2 and an increase in proinflammatory macrophages M1 that release cytokines such as tumor necrosis factor-α (TNF-α). Adenosine (Ado) cannot be converted to inosine (Ino) and binds to its receptors in neutrophils, leading to neutrophil extracellular trap formation, which induces TNF-α release from M1 macrophages. When ADA2 activity decreases, deoxyadenosine (dAdo) concentration increases. dAdo can enter the cell, and is converted to dIno by ADA1, thereby inhibiting the synthesis of SAM and the transmethylation reactions. The reduction in the activity of DNA methyltransferases (DNAMT) leads to the expression of endogenous retroviral elements that result in increased transcription of IFN-β (interferon β). ENT1: equilibrative nucleoside transporter 1. Parts of the figure were drawn by using pictures from Medical gallery of Blausen Medical [257]. Medical gallery of Blausen Medical is licensed under a Creative Commons Attribution 4.0 Unported License (https://creativecommons.org/licenses/by/4.0/ accessed on 23 May 2023).

Zhou et al. [243] showed that monocytes from ADA2-deficient patients could differentiate into pro-inflammatory M1 macrophages but not anti-inflammatory M2 macrophages. Some clinical manifestations of ADA2 deficiency have been reproduced in the zebrafish model. The knockdown of zebrafish ADA2 caused intracranial haemorrhages and neutropenia. This phenotype was prevented by co-injection with non-mutated human ADA2 [243]. The same authors also observed that monocytes from ADA2-deficient patients induced damage in co-cultured endothelial cells [243]. In addition, the observed vasculopathy may be derived, at least partially, from neutrophil extracellular trap formation, induced by adenosine signaling (Figure 5) [251,252]. Neutrophil extracellular trap formation is a form of cell death characterized by the extracellular release of granule proteins bound to a decondensed chromatin reticulum. Neutrophil extracellular traps are formed both in response to invaders and to endogenous inflammatory signals through downstream intracellular mediators that include reactive oxygen species. Neutrophil extracellular traps can confine and facilitate the elimination of various pathogens, but they are also involved in immune-mediated pathologies. Neutrophils release pro-inflammatory mediators during trap formation and when inflammatory signaling persists for long periods, the aggregation of the traps can lead to vascular occlusions [253]. Moreover, some manifestations of ADA2 deficiency could result from the increased production of inflammatory cytokines such as TNF-α by pro-inflammatory M1 macrophages [229], and from the type I/type II interferon (IFN) pathway upregulation in ADA2-deficient T cells and monocytes, as well as from increased IFN-β secretion directly from endothelial cells [254]. Indeed, reduced ADA2 activity is associated with the entry of deoxyadenosine into the endothelial cells, where it is converted by ADA1 into deoxyinosine. This intracellular increase in deoxyinosine can inhibit methionine adenosyl transferase [255], thus interfering with the cellular methionine cycle, leading to hypomethylation and the overexpression of immune-stimulatory endogenous retroviral elements and thereby IFN-β production [255] (Figure 5). Moreover, the transcriptomic and proteomic analyses of peripheral blood mononuclear cells from ADA2-deficient patients have shown the upregulation of type II IFN signaling, including the signal transducer and activator of transcription 1 hyperactivation compared with controls [256]. Altogether, these observations suggest that ADA2 deficiency results in profound immunological dysregulation characterized by systemic inflammation and the inappropriate induction of cytokines.

Recent studies suggest that impairment in ADA regulation or isoenzyme distribution is associated with neurodegenerative diseases such as amyotrophic lateral sclerosis (ALS) and multiple sclerosis [224,258,259], although the mechanism leading to neurodegeneration is not clear at the moment. A reduction in ADA activity has been found in astrocytes and fibroblasts of the patients of the C9orf72 type of ALS and in astrocytes of sporadic ALS patients. Adenosine-induced toxicity was higher in astrocytes derived from patients, and this toxic effect was also found when ADA inhibitors were added to cell lines. The addition of inosine increased motor neuron survival in cocultures with induced astrocytes [258], suggesting that inosine supplementation might be considered as a therapeutical tool in ALS patients. Kutryb-Zajac et al. [259] have found an increased ratio of ADA1/ADA2 in both the plasma and the cerebrospinal fluid of multiple sclerosis patients. The authors have proposed that the increased ADA1/ADA2 ratio in patients may lead to ADA1-mediated proinflammatory processes and to a decrease in ADA2-dependent neuroprotective effects, promoting multiple sclerosis [259].

## 10. S-Adenosylhomocysteine Hydrolase

SAHH catalyzes the reversible hydrolysis of SAH to adenosine and L-homocysteine (Figure 1 and Figure 4). Thus, it regulates the intracellular SAH concentration thought to be important for transmethylation reactions. SAHH has been shown to have a role in various cellular functions through the regulation of methylation reactions, including epigenetic homeostasis, stem cell and cancer cell proliferation, and circadian clock [260,261,262,263]. The deficit of SAHH, one of the different causes of hypermethioninemia, is an autosomal recessive disorder that was first reported in Gaull et al. [264] and Labrune et al. [265] and received molecular confirmation in 2004 [266]. To date, only 16 patients with this disorder have been reported [267]. Patients with SAHH deficiency present developmental delay and hypotonia due to myopathy with markedly increased creatine kinase plasma levels; cerebral hypomyelination, coagulation abnormalities, and hepatopathy are more variable. Microcephaly, strabismus, and behavioral changes are frequent [217,267]. Two individuals, dead in early infancy, were reported to have fetal hydrops, congenital brain anomalies (pontine and cerebellar hypoplasia, hypoplastic corpus callosum), liver failure, and respiratory insufficiency due to severe muscle weakness [268]. The disease is typically severe, but the phenotype can also be milder and one pre-symptomatic case has been reported [269]. Hepatocellular carcinoma was reported in one patient [269].

### 10.1. Diagnosis and Treatment

Specific biochemical findings include markedly increased plasma SAH and SAM in combination with normal or near normal total homocysteine and hypermethioninemia, which is not always present, particularly not in early infancy. A reduced methionine intake, sometimes with supplementation with phosphatidylcholine and creatine, has been tried in several patients [267,269,270,271,272]. SAM and SAH levels decreased but remained higher than normal, creatine kinase and liver function were not modified, and only some patients showed clinical improvements in muscle strength and mental alertness. However, the developmental delay persisted [267]. It has been suggested that early treatment improves the outcome [270,271].

### 10.2. Proposed Mechanistic Basis

It has been proposed that some pathological manifestations could be ascribed to choline depletion as a result of the inhibition of the methyltransferase that converts phosphatidylethanolamine into phosphatidylcholine, which is a precursor of choline. Choline and phosphatidylcholine are important in the function of liver, muscle, and the nervous system. Choline depletion affects muscle membrane lipid composition and intracellular lipid metabolism and causes the accumulation of lipid droplets in muscle cells [273], but no lipid accumulation has been found in the muscles of three patients [272], suggesting that choline depletion does not have an important role in the muscular manifestations of these patients. The inhibition of guanidinoacetate methyltransferase could result in creatine depletion and increased guanidinoacetate levels. Indeed, the increased concentration of guanidinoacetate in plasma or urine has been reported in patients [266,274], suggesting the potential usefulness of creatine supplementation. There is no animal model for SAHH deficiency: embryo mice homozygous for a deletion overlapping the SAHH gene die around the time of implantation [275], suggesting that the complete loss of the gene is lethal. It is possible that, in addition to the effects described above, the inhibition of DNA methyltransferases could lead to abnormal gene methylation and expression, leading to pathological changes already present in utero.

## 11. Purine Nucleoside Phosphorylase

Human PNP (hPNP) catalyzes the phosphorolysis of 6-oxo-ribo and 6-oxo-2′-deoxyribo nucleosides [276]. Differently from the *Escherichia coli* enzyme, hPNP does not act on adenosine and deoxyadenosine [277], which must be firstly deaminated by ADA and in turn catabolized by hPNP [278] (Figure 1). PNP deficiency is a rare, autosomal-recessive disorder, caused by loss-of-function mutations in the *hPNP* gene, characterized by severe T-cell immunodeficiency [279,280], accompanied in two thirds of the cases by neurologic symptoms, and sometimes appearing before any infectious problem [280]. They include ataxia, developmental delay, and intellectual disability [10,281,282]. Autoimmune manifestations, such as idiopathic thrombocytopenic purpura, thyroiditis, and lupus are reported in one third of the cases [283,284]. Symptoms may appear at different ages, typically from four months to six years of age, and their severity may be extremely variable. A clear genotype–phenotype correlation has not been established so far, although 8–11% PNP residual activity has been reported to be the minimum required for near-normal immunity and typical neurological development in humans [285].

### 11.1. Diagnosis and Treatment

Hallmark diagnostic markers of PNP deficiency include increased urine or blood levels of inosine, guanosine, and their deoxy forms. Lower levels of urine and serum UA are also usually found in patients. Recurrent infections and lymphopenia are a common finding, with a SCID pattern. An early diagnosis in the preinfection period would be helpful in allowing for the most effective therapeutic treatment. In this regard, to identify patients with PNP deficiency, both HPLC and tandem mass spectrometry methods [286,287] were devised to quantify purine metabolites on dried blood spots. These low-cost methods have been demonstrated to be valuable for the diagnosis of PNP-deficient patients at birth but, to our knowledge, have not been included in routine newborn screening programs. The individuation of the disorder at birth would allow for an early diagnosis and an early treatment with hematopoietic stem cell transplantation (HSCT), which so far represents the only curative treatment for this disorder [288,289]. After a HSCT intervention, patients may achieve successful immune reconstitution and are free from infections; however, the neurological status is not fully resolved [288,289,290].

### 11.2. Proposed Mechanistic Basis

Among purines accumulating in cases of PNP deficiency, only deoxyguanosine (dGuo) can be phosphorylated both in cytoplasm by deoxycytidine kinase (dCK) [291] and in mitochondria by dGuo kinase (dGK) [292]. Deoxyguanosine monophosphate is then converted into dGTP, which has been hypothesized to be the cytotoxic compound [14]. dGTP, which is an allosteric modulator of ribonucleotide reductase, interferes with cell replication and appears to be lethal for T-lymphocytes. PNP-KO mice, obtained by knocking out the *Pnp* gene, were used by Papinazath et al. [292] to demonstrate that the mitochondrial apoptotic pathway was significantly increased in CD4/CD8 double-positive thymocytes, and that this increase was dGuo-dependent. At birth, in many PNP-deficient patients [280], as with PNP-KO mice [292], T-cell production is normal, but the gradual accumulation of toxic purine compounds results in the progressive damage of the thymus. This is a strong indication that an early identification and treatment might favor the proper development of the thymus in patients affected by PNP deficiency.

**Figure 6:**
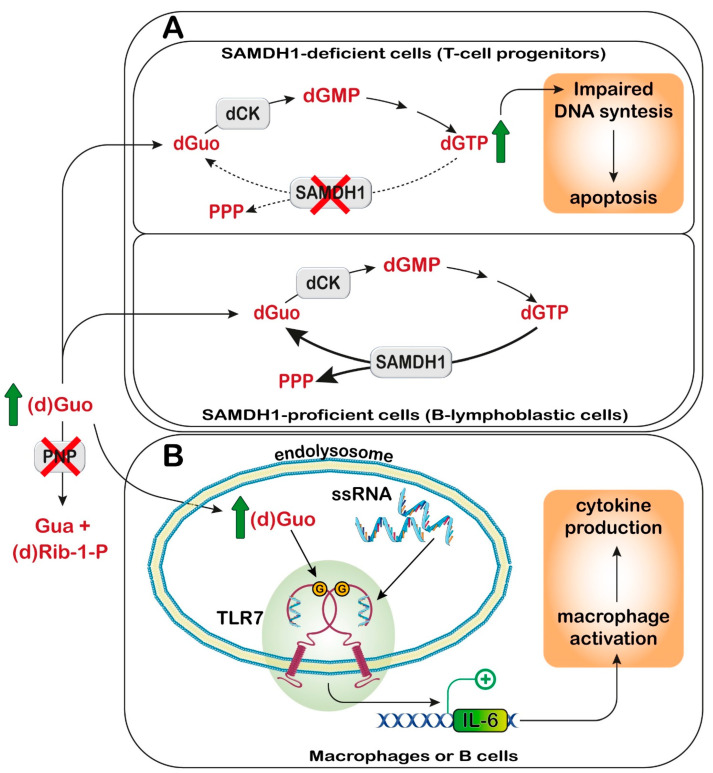
Immunological outcomes of purine nucleoside phosphorylase (PNP) inhibition. The lack of PNP causes accumulation of both ribo and deoxyribo guanosine [(d)Guo]. Panel (**A**), upper: Deoxyguanosine (dGuo) is phosphorylated by deoxycytidine kinase (dCK) to deoxyGMP (dGMP), then converted to deoxyGTP (dGTP), which accumulates in sterile alpha motif and HD domain containing protein 1 (SAMHD1)-deficient cells (namely, T-cell progenitors). Panel (**A**), lower: In SAMDH1-proficient cells (namely, B lymphoblastic cells), dGTP does not accumulate because it is efficiently converted to dGuo and triphosphate. Panel (**B**): Elevated endolysosomal guanosine nucleoside levels trigger Toll-like receptor 7 (TLR 7) activation, through a dual binding of single-stranded ribonucleic acid (ssRNA) and guanosine, which causes transcription of the gene encoding for interleukin-6 (IL-6). Parts of the figure were drawn by using pictures from Servier Medical Art. Servier Medical Art by Servier is licensed under a Creative Commons Attribution 3.0 Unported License (https://creativecommons.org/licenses/by/3.0/ accessed on 23 May 2023).

More recently, Abt et al. [291] have focused their attention on the paradoxical and still not fully understood association of PNP deficiency with both immunodeficiency and autoimmunity. To mimic thymopoiesis, Abt et al. [291] utilized the human thymic organoid model [293], supplemented with 5 µM dGuo, a concentration similar to that found in PNP-deficient patients [287], in the presence or absence of a powerful and selective PNP inhibitor. PNP inhibition caused a developmental block, evidenced by CD4/CD8 double-negative accumulation and a significant decrease in the organoid cellularity. Differently from Papinazath et al. [292], who identified dGK as the enzyme necessary for the phosphorylation of dGuo, Abt et al. [291] indicated dCK as the enzyme responsible for dGuo salvage. In fact, the noxious effect of PNP inhibition was completely rescued by a selective and potent inhibitor of dCK. The accumulated dGTP causes the inhibition of DNA synthesis and impaired pyrimidine dNTP synthesis, thus triggering the apoptotic pathway. The same authors underline a central role played by sterile alpha motif and HD domain containing protein 1 (SAMHD1) for the cellular lethality of PNP inactivation [291]. SAMHD1 catalyzes the hydrolysis of dNTP to triphosphate and respective 2′deoxynucleosides, and is essential for the regulation of the dNTP pool [294]. Abt et al. [291] demonstrated that acute lymphoblastic leukemia cells (T-ALL) (Figure 6A, upper), differently from other immune lineages (such as B-lymphoblastic cells or acute myeloid leukemia cells) (Figure 6A, lower), were sensitive to PNP inhibition, with a loss of cell viability and the activation of the caspase cascade. In fact, T-ALL cells and human thymocytes possess low levels of SAMHD1, and therefore cannot efficiently hydrolyze the excess dGTP, which might explain their selective sensitivity to PNP inactivation. In addition to T-cell mediated immunodeficiency, PNP deficiency is often associated with various autoimmune and inflammatory phenotypes in humans [280]. To elucidate the mechanisms responsible for this phenotype, Abt et al. [291], using a C57BL/6 mouse model, bone-marrow derived macrophages, and other cellular models, demonstrated that PNP inhibition caused a robust increase in interleukin-6 (IL-6), a cytokine associated with autoimmunity and inflammation [295]. This effect was independent of dCK, since the inhibition of the enzyme could not reverse the alterations induced by PNP inactivation and appeared to be dependent on Toll-like receptor 7 (TLR 7) activation [291]. TLR7 is an endosomal innate immune sensor capable of binding guanine nucleosides and single-stranded ribonucleic acid (ssRNA) [296]. The dual binding of ssRNA and guanosine triggers TLR7 dimerization, with the activation of downstream signaling. Therefore, PNP inactivation allows for guanine nucleoside accumulation, facilitating their transit to endolysosomes and their binding to TLR7, thus starting the signaling pathway, with an increased expression of IL-6 [291] (Figure 6B). IL-6 has been reported to promote the development of spontaneous germinal centers in secondary lymphoid tissues, associated with autoimmunity and autoinflammation [295]. These data offer a new important translational perspective, adding dCK and TLR7 signaling as possible pharmacological targets for a therapeutic strategy of PNP deficiency.

In addition to severe immunodeficiency and autoimmunity, PNP-deficiency is often associated with neurological symptoms of still unknown etiology. The neurological abnormalities often manifest before the immune defects and this suggests a non-immune mechanism [297]. PNP-KO mice suffer not only from immune abnormalities, but also manifest motorneuron defects, with a reduced number of cerebellar Purkinje cells [298]. The treatment of PNP-KO mice at birth with PNP replacement prevented the development of neurological defects [298], while initiation of the treatment at 4 weeks was unsuccessful, likely because irreversible brain injury had already occurred. In contrast, the enzyme replacement in PNP-KO mice corrected the metabolic disorder and the immune defects, even when treatment was initiated at 3–4 weeks of age [299], possibly reflecting an irreversible toxicity to non-renewable neurons versus the renewable thymocytes. Recently, Tsui et al. [300] have used neurons derived from the iPSC of PNP-deficient patients as a tool to understand the mechanisms for the human neuromotor abnormalities. The PNP-deficient neurons exhibited increased spontaneous apoptosis, evidenced by increased cleaved caspase-3 and -9, together with a decreased mitochondrial membrane potential [300], a mechanism similar to that described in mouse thymocytes [292]. Moreover, p53 expression was elevated and its inhibition with pifithrin-α prevented apoptosis [300]. Tsui et al. [300] proposed an apoptotic mechanism in which dGuo was phosphorylated by mitochondrial dGK or cytoplasmic dCK, thus resulting in an increase in dGTP, which, as described before, impairs ribonucleotide reductase activity, with dNTP unbalance, damage in DNA synthesis, and the induction of p53 expression [301]. In turn, p53 induces caspase activation through mitochondrial cytochrome c release [302], with the subsequent activation of the caspase cascade.

## 12. Discussion

This paper tried to gather important aspects of inborn errors in purine salvage and breakdown causing metabolic disorders, often resulting in nervous system diseases with diverse manifestations and apparently different etiology. Table 1 summarizes the pathological, clinical, and diagnostic aspects related to the defects of the enzymes under consideration. A reappraisal of the available knowledge about these rare diseases leads to observe that taken together they often show similarities and metabolic connections, which might draw closer to the understanding of the pathogenesis of neurological anomalies. GTP depletion is a common feature encountered in different disorders (namely, in cases of HPRT, AMPD2, and IMPDH2 deficiencies, and PRPS superactivity) and has been suggested to lead to alterations of the GTP-dependent synthesis of BH4, or to defective GTP-dependent initiation of protein translation, or else to altered signal transduction through G-proteins. An accumulation of specific metabolites, such as dGTP or dATP (in PNP and ADA deficiencies), known to modulate the activity of ribonucleotide reductase, or an unbalance in nucleotide levels (in cN-II loss-of-function), might result in impaired DNA synthesis, often triggering an apoptotic program. Abnormal levels of purine bases or nucleosides (found in HPRT, PNP, ADA, and ADK deficiencies and PRPS superactivity) might interfere with the trafficking of adenosine, and therefore with the purinergic signaling, whose involvement in neurodevelopment disorders is now well documented [303]. Moreover, the AMPK/mTOR signaling pathway should be taken into account when the affected enzymes are essential for the regulation of AMP levels (cN-I and cN-II loss-of-function variants and AMPD and ADK deficiency) or in HPRT deficiency.

Inborn errors of purine metabolism are considered rare diseases, but the difficulty of diagnosis, depending on many factors, might prevent their correct identification. Mutations may affect different aspects of the enzyme functions, such as the kinetic parameters, or the binding of other regulating proteins or small molecules, leading to a panel of alterations in enzymatic activity and therefore to different symptoms. A complete lack of an enzyme activity may compromise vital functions resulting in life threatening diseases. Partial enzyme dysfunction may result in symptoms easily misdiagnosed as one of the many infantile syndromes characterized by neuropsychiatric, neuromotor, and neurosensorial impairments. In this regard, using metabolomic and transcriptomic approaches, dysfunctions of purine metabolism, other than the well-known ADSL deficiency, have been reported in autism spectrum disorders [304,305], such as a significant increase in ADA activity, with a reduction in UA [304], and an increase in hypoxanthine, inosine, and xanthosine [305]. Another reason for misdiagnosis is the occurrence of compensatory mechanisms, which may differ among individuals and may attenuate the effects of the purine inborn error, giving rise to various phenotypes that do not completely correlate with the genotype.

Over the years, several cell models have been developed and employed to explore the metabolic features and investigate the molecular mechanisms underpinning these rare diseases. Most studies have been initially conducted using easily available cells, such as erythrocytes and cultured fibroblasts, isolated from patients for diagnostic purposes. Other cellular models were also studied, but they quickly revealed their limitations: failing to recapitulate the different enzymatic expression or activity in different tissues or cell types, in different stages of development, or lacking several relevant pathways (e.g., protein synthesis machinery in erythrocytes). Furthermore, most of the pathologies of still unknown etiology, associated with purine inborn errors, concern the nervous system. The pathophysiology of neurological defects cannot be studied directly in the patients, because of the ethical implications of obtaining samples of brain tissue. Therefore, animal models were developed as an alternative approach, which, unfortunately, often did not completely reflect the human phenotype and failed to address many of the unresolved neurological features. In this scenario, the development of iPSC technology strongly changed the modelling and analysis of human neurologic diseases in vivo. In recent years, iPSCs have been used to generate disease-relevant and patient-specific neuronal cell types, providing new tools to prompt the understanding of the pathophysiology of several neurologic disorders as well as the discovery and development of novel therapies. Altogether, all experimental approaches indicate that the noxious effects caused by the enzyme loss- or gain-of-function occur during neurodevelopment, leading to the conclusion that only very early interventions might be effective.

Finally, we reiterate the relevance of newborn screening programs, which at present do not represent routine clinical practice. The experiences of the early detection of SCID, using the T-cell excision circle assay technique [306,307,308,309], represent a remarkable exception. The usefulness of neonatal screening can be questioned by the observation that some of the effects of the disorders may present during organogenesis, including that of the nervous system. However, several affected individuals appear normal at birth [86,310,311], suggesting a protective effect of the transplacental maternal–fetal blood exchange.

Overall, the extreme rarity of the described diseases should be reconsidered in light of reports obtained through the more sophisticated investigation techniques now available.

## 13. Conclusions

The analysis of the metabolic profiles of patients in many cases provides an alarm bell for the suspicion of several inborn errors of purine metabolism. For an early detection of some of them, the determination of UA levels in blood and/or urine, a simple and low-cost analysis, can be a very useful tool. This diagnostic approach has recently been suggested by Jurecka and Tylki-Szymanka [8], who have also outlined some pitfalls in the use of UA as a diagnostic marker. For the diagnosis of the diseases in which UA alterations can be excluded, the identification of other altered metabolite amounts can be determined. For this purpose, low-cost HPLC and tandem mass spectrometry methods have been devised to quantify purine metabolites on dried blood spots. Finally, genetic analysis, which is currently not too difficult to obtain, may be definitive for correct diagnoses. Someone might question the usefulness of an early diagnosis, since no appropriate therapy is available for many of these diseases. Nevertheless, should the disease result from the toxic effect of a specific metabolite on the proliferation, differentiation, and migration of neural progenitors, an effort can be made to keep its concentration under control, at least during the initial phases of life. In this light, the inclusion of these so-called rare diseases in newborn screening programs could be beneficial.

## Figures and Tables

**Table 1:** Pathological, clinical, and diagnostic features of inborn errors of purines.

Enzyme (Name; Symbol; EC)	Gene (Symbol; Location; Omim Code)	Pathology (and Variants)	Clinical Features	Diagnostics
Phosphoribosylpyrophosphate Synthetase; PRPS1; EC 2.7.6.1	*PRPS1*; Xq22.3; OMIM 311850	PRPS superactivity	Stones; gout Sensorineural deafness Neuromotor retardation	Hyperuricemia and/or hyperuricuria High PRPS activity in red cells and fibroblasts High PRPP levels; low GTP and NAD levels in red cell Genetic analysis
		PRPS deficiency(DFNX1; CMTX5; Arts syndrome)	Hearing impairment Neuromotor retardation Optic atrophy	Normal serum UA Low to absent PRPS activity in red cells and fibroblasts Genetic analysis
Hypoxanthine-Guanine Phosphoribosyltyransferase; HPRT; EC 2.4.2.8	*HPRT*; Xq26.2-q26.3; OMIM 308000	Lesch–Nyhan Disease (total deficiency)Lesch–Nyhan variants (partial deficiency)	Gouty arthritis Neurobehavioral disturbance Lithiasis Megaloblastic anemia	Hyperuricemia and/or hyperuricuria Absent to very low HPRT activity in red cells, lymphocytes, or fibroblasts Genetic analysis
Adenylosuccinate lyase; ADSL; EC 4.3.2.2	*ADSL*; 22q13.1; OMIM 608222	ADSL deficiency S-Ado/SAICAr < 1 in fatal neonatal form S-Ado/SAICAr = 1 in severe type I S-Ado/SAICAr >1 in moderate type II	Fatal neonatal form: hypokinesia, intractable seizures, and respiratory failure Severe type I form: severe psychomotor retardation, microcephaly, seizures, autistic features Moderate type II form: moderate or slight psychomotor retardation	High levels of SAICAr and S-Ado in urine, plasma, and CSF Low ADSL activity (>2%) in fibroblasts Genetic analysis
Inosine-5′-monophosphate dehydrogenase IMPDH; EC 1.1.1.205	*IMPDH1*; 7q32.1; OMIM 146690	IMPDH1 malfunction: Retinitis pigmentosa type 10 Leber congenital amaurosis type 11	Retinal degeneration Night blindness Loss of peripheral visual field	Genetic analysis
	*IMPDH2*; 3p21.31; OMIM 146691	IMPDH2 deficiency: Severe juvenile neuropathies	Dystonia Tremor	Genetic analysis
Ectosolic 5′-nucleotidase; eN; EC 3.1.3.5	*NT5E*; 6q14.3; OMIM 129190	eN hyperactivity: Nucleotidase-associated pervasive developmental disorder (NAPDD)	Hyperactivity, impulsiveness, aggressiveness Poor attention span and social interaction Neurological symptoms: seizure ataxia, impaired fine motor control	eN activity in fibroblasts Hypouricuria
		eN deficiency: Calcification of joints and arteries (CALJA)	Calcification of joints and arteries	Genetic analysis
Cytosolic 5′-nucleotidase; cN-II; EC 3.1.3.5	*NT5C2*; 10q24.32-q24.33; OMIM 600417	Hereditary spastic paraplegia type 45 (HSP-cN-II deficiency)	Loss of corticospinal motor tract function Progressive limb spasticity and weakness	Genetic analysis
Adenosine monophosphate deaminase; AMPD; EC 3.5.4.6	*AMPD1*; 1p13.2; OMIM 102770	Myoadenylate deaminase deficiency (AMPD1)	Exercise induced early fatigue, cramps, and myalgia Hypersomnia	Low AMDP1 activity in skeletal muscles AMDP immunohistochemistry in muscle biopsy Genetic analysis
	*AMPD2*; 1p13.3; OMIM 102771	Pontocerebellar hypoplasia type 9 (AMPD2 deficiency)	Microcephaly Hypoplastic/absent corpus callosum Severe intellectual disability	Low AMPD2 activity Low AMPD2 levels Genetic analysis
Adenosine kinase; ADK; EC 2.7.1.20	*ADK*; 10q22.2; OMIM 102750	ADK deficiency	Liver dysfunction Delayed psychomotor development Mild dysmorphic features Hypotonia and epilepsy Vascular abnormalities	Hypermethioninemia High levels of S-adenosylmethionine and S-adenosylhomocysteine Genetic analysis
Adenosine deaminase; ADA; EC 3.5.4.4	*ADA1*; 20q13.12; OMIM 608958	Severe combined immunodeficiency (SCID-ADA1 deficiency)	Lymphocytopenia Recurrent infections Neurodevelopmental deficits Sensorial deafness Skeletal abnormalities	High levels of dAdo, dATP, total dAdo nucleotides Absent to extremely low ADA1 activity. Genetic analysis
	*ADA2*; 22q11.1; OMIM 607575	ADA2 deficiency	Autoinflammatory, vasculopatic, hematologic and immune system dysfunction Neurological manifestations (dysartria, ataxia, palsy) Cognitive impairment Neuropathy (central and peripheral) Intracerebral haemorrhaging and aneurysm Immunodeficiency	Absent to low ADA2 activity in plasma and serum Genetic analysis
S-Adenosylhomocysteine hydrolase; SAHH; EC 3.3.1.1	*AHCY*; 20q11.22; OMIM 180960	SAHH deficiency	Developmental delay Hypotonia Cerebral hypomyelination, coagulation abnormalities and hepatopathy (variable) Microcephaly, strabismus and behavioral changes (frequent)	High levels of SAH and SAM in plasma Hypermethioninemia Normal homocysteinemia Increased activity of creatine kinase Genetic analysis
Purine nucleoside phosphorylase; PNP; EC 2.4.2.1	*PNP*; 14q11.2; OMIM 164050	PNP deficiency (SCID)	T-cell immunodeficiency Neurological symptoms Recurrent infections Autoimmuny (idiopathic thrombocytopenic purpura, thyroiditis, SLE) Developmental delay	Hypouricemia and/or hypouricuria High levels of inosine, guanosine (and their deoxy forms) in urine and plasma Low PNP activity in red cells Genetic analysis
